# Ethnobotany in Rayones, Nuevo León, México

**DOI:** 10.1186/1746-4269-10-62

**Published:** 2014-09-01

**Authors:** Eduardo Estrada-Castillón, Miriam Garza-López, José Ángel Villarreal-Quintanilla, María Magdalena Salinas-Rodríguez, Brianda Elizabeth Soto-Mata, Humberto González-Rodríguez, Dino Ulises González-Uribe, Israel Cantú-Silva, Artemio Carrillo-Parra, César Cantú-Ayala

**Affiliations:** 1 Universidad Autónoma de Nuevo León, Linares, Nuevo León, Mexico; 2 Universidad Autónoma Agraria Antonio Narro, Saltillo, Coahuila, Mexico

**Keywords:** Ethnobotany, Medicinal, Food, Construction, Ceremonial, Ornamental species, Rayones, Nuevo León, México

## Abstract

**Background:**

Trough collections of plants and interviews with 110 individuals, an ethnobotanical study was conducted in order to determine the knowledge and use plant species in Rayones, Nuevo Leon, Mexico. The aim of this study was to record all useful plants and their uses, to know whether differences exist in the knowledge about the number of species and uses between women and men, and to know if there is a correlation between the age of individuals and knowledge of species and their uses.

**Methods:**

A total of 110 persons were interviewed (56 men, 56 women). Semistructured interviews were carried out. The data were analyzed by means of Student *t* test and the Pearson Correlation Coeficient.

**Results:**

A total of 252 species, 228 genera and 91 families of vascular plants were recorded. Astraceae, Fabaceae and are the most important families with useful species and *Agave* and *Opuntia* are the genera with the highest number of useful species. One hundred and thirty six species are considered as medicinal. *Agave*, *Acacia* and *Citrus* are the genera with the highest number of medicinal species. Other uses includes edible, spiritual rituals, construction and ornamentals. There was a non-significant correlation between the person’s age and number of species, but a significant very low negative correlation between the person’s age and number of uses was found.

**Conclusions:**

Knowing their medicinal uses is an important issue for the people of Rayones. Boiling and preparing infusions are the main ways of using plants by residents. The leaves, the branches, and the fruits are the most commonly used parts. Almost 18% of the flora is used for wood and construction purposes. Several uses such as cosmetic, shampoo, firming skin tonics and health hair products recorded in Rayones has not been reported for other areas in the state of Nuevo León. In Rayones, women have a greater knowledge about plants and their uses than men, particularly, medicinal plants, but, men have a greater knowledge about wood and construction species.

## Background

The use of plants over time has allowed a better understaning of their properties in virtually all societies. Our knowledge of plants today is the result of the historical legacy of our ancestors, who learned empirically by trial and error, coupled with the new scientific knowledge used today to find new uses for them. The medicinal plants are a therapeutic resource for excellence in Mexican traditional medicine and can be an important element in the implementation of new health plans, combining traditional and scientific knowledge [1]. In many cases and in different societies, the age is a factor highly correlated with the number and uses of plants known by individuals [2-6], the older the individual, the greater the knowledge of plants and their uses they have. Several studies have shown that men know a greater number of useful plant species than women [7-12], other studies have shown that the number of plants is not always correlated with gender (female–male) [13], but this does correlate with specific uses of certain species [6,14-18] such as medicinal and edible plants [7,8,19,20]. Men and women in rural areas inhabited by scrublands of northern Mexico use many wild and cultivated plants for different purposes such as medicinal, food, forage, wood, charcoal, and construction. In this work, we chose to work in Rayones county, since much of the knowledge of useful plants in arid areas of northeastern Mexico is found in rural areas. The municipality of Rayones is located in a semi-arid area with a rich tradition of using plants. The focus of this study is to increase the documented knowledge concerning to useful plants in semiarid region of northeastern Mexico, and to determine what sector of the population uses more species in order to understand if their use is maintained or decreases among them, and, 1) to know if there are differences between women and men in the knowledge of number of plants and the number of uses, and 2) to know if the age of the people determines the number of species and the number of uses known. Our hypotheses are 1) women know a greater number of plants and uses than men (according to our previous experience in the area [11,12]. Moreover, we wanted to address 2) the older people know a greater number of species and a greater number of uses than their younger counterparts.

## Methods

### Study area

**Figure 1:**
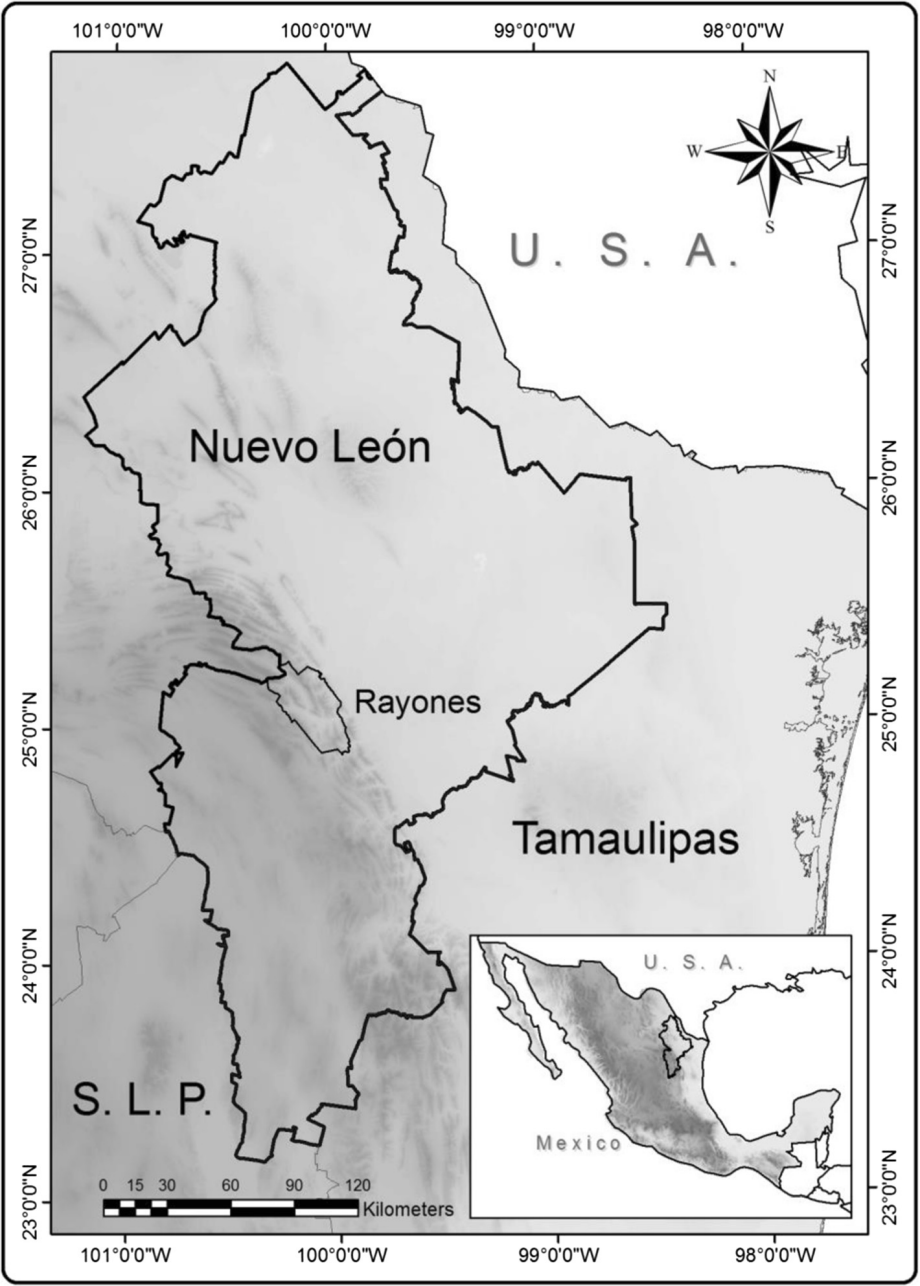
Study area, state of Nuevo León showing the location of Municipality of Rayones and adjacente States.

The municipality of Rayones is located in the semiarid central part of the state of Nuevo León, most of its surface is found into narrow valleys with rough topography. The mountains are one of the main sources of natural resources for the Rayones residents. Several small rivers originate here and their water irrigates several species of plants of economic importance, such as hickory (*Carya illinoinensis*), avocado (*Persea americana* var. *drymifolia*), membrillo (*Cydonia oblonga*), pomegranate (*Punica granatum*), figs (*Ficus carica*), corn (*Zea mays*), among others. There is a rich wild plant diversity used by residents in several ways. The total population in Rayones municipality comprises 2628 persons [21]. Nearly 75% of the population has some kind of government or private health assistance. Rough mountains, huge cliffs, deep canyons, and permanent rivers make Rayones an attractive place for rappelling, hiking, swimming, camping and other leisure activities. Most of the cultivated areas available in Rayones have *Carya illinoiensis* for pecan production. In the absence of modern industry, residents look for alternatives to earn money and make a living such as selling groceries, cattle, and cultivated fruits. Some people (women and men) sell wild dried plants. Rayones Valley is characterized by arid and arid-temperate climates, located into the transitional border between Sierra Oriental physiographic province [20] and the Chihuahuan Desert Ecoregion [22]. Rayones has an area of 905 km^2^, and is within the Gran Sierra Plegada physiographic subprovince [23], 24°52′40″-25°15′18″ N and 99°55′35″-100°19′04″ W (Figure 1). The valleys are dominated by shallow soils and rendzinas while the mountains’ dominant soils are lithosol associations with calcaric rhegozol, dark rendzinas, calcaric feozem, chromic luvizol, and haplic xerosols. The vegetation is mainly composed by scrublands and forest. There are two types of scrublands, the rosetophyllous scrub, where *Agave*, *Yucca, Dasylirion* and *Hechtia* are the main genera, and the piedmont scrub, dominated by *Helietta parvifolia, Havardia pallens, Fraxinus greggii, Forestiera angustifolia, Acacia rigidula, Disospyros texana,* and *Neorpinglea integrifolia*[23]. The forest is composed by two main associated genera *Quercus-Pinus.* The climate of the valleys and low altitudes belongs to the BS type [23], the average annual temperature is 21°C and, the mean annual precipitation is 693 mm, while in mountains the climate belongs to the C type [23], the average annual temperature is 16°C and, the mean annual precipitation is 755 mm, and the altitudes oscillates from 890–1560 m [23].

### Field work

During 2012 (from March to December) all wild and cultivated species present in the study area were collected and digital photographs of each species were taken. The samples were pressed, dried, and identified by the authors. A printed catalog was made with the species photographed to show to the residents. Voucher specimens of each plant species was pressed, dried, and identified by the authors and deposited together with all the relevant data in the herbarium (CFNL, Linares, N. L.).

### Interviews

Information on the number of species and number of uses and common names were obtained from 110 residents interviewed from March-July 2012 (50 interviews) and July-December 2012 (60 interviews). Ethnobotanical information was collected through semi-structured interviews [24], and according to the efficiency decreasing law [12]. Several questions were asked, a) what is it used for (medicinal, food, forage, wood, charcoal, construction, ceremonial, etc.), b) how is the plant used (depending of its use, raw, boiled, macerated, toasted), c) what parts of the plant are used. Moreover, the printed catalog was shown to the interviewed residents in order to inquire the use of each species. Additionally, we made several field trips with some men and women living in the area for *in situ* identification of some uncommon plants when we had questions regarding their identification. The age of the residents being interviewed ranged from 20–92 years old, 59% of them are 56 years old or older, and 41% of them are 55 years old or younger. According our experience in previous works in this State [11,12]; we skewed our interviews number, and we interviewed mainly people older than 32 years old or older instead of younger people, since we have seen that older people know more about plants and their uses. All interviews were carried out in the scrublands area, since all people live there and none of the respondents live in the temperate woods. All the people that were interviewed were born in this area or had been residents of this area for at least 32 years. All the interviews were performed in Spanish, since none of informants speaks a different dialect or language; none of the respondents belong to a different ethnic group. All information concerning plants names and their uses were recorded in notes and photographs.

### Data analysis

Considering gender as independent group, we compare each variable (number of species and number of uses) between men and women by using the *t* Test [25]. For question 2, age of individuals was correlated with the number of species and the number of uses by means of the Pearson Correlation Coefficient [25,26].

## Results

### Knowledge of plants and uses

**Figure 2:**
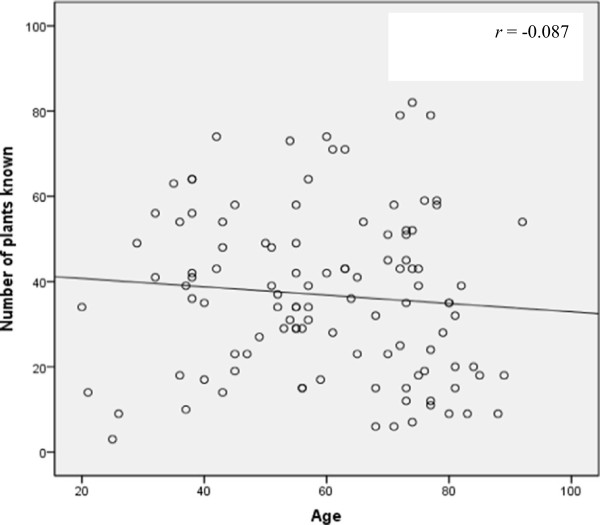
**Correlation between age and number of plants for residents in Rayones, Nuevo Leon, Mexico.** There was no significant correlation between variables.

**Figure 3:**
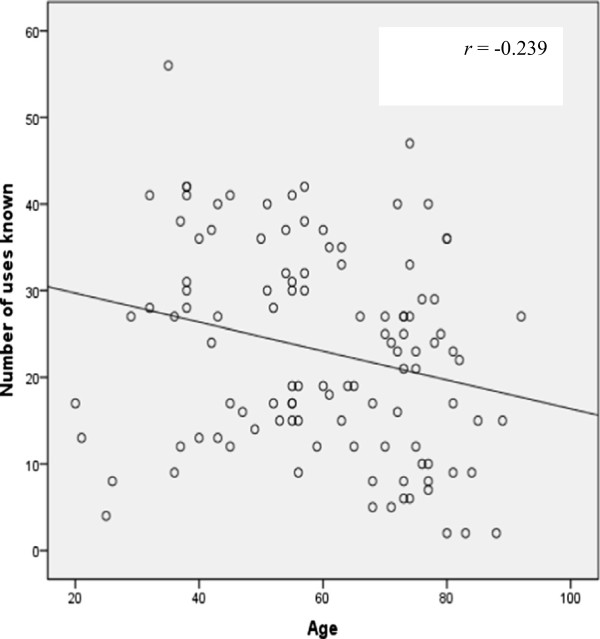
**Correlation between age and number of uses for residents in Rayones, Nuevo Leon, Mexico.** There was a very low significant negative correlation between variables.

As in southern Nuevo León [12], women from Rayones know more plants and uses than men. In average, women mentioned 50 plants and 36 uses while men mentioned 36.5 plants and 24.5 uses. The women know greater number of wild medicinal plants and their uses than men, but men, know more wild timber species and their uses than women. Men and women know almost the same number of cultivated and wild food plants. In average, women mentioned 19 food plants, while men mentioned 16 species. The age was not correlated with the knowledge of species and their uses, however, it is interesting to note that the youngest (20–31 years old) and the oldest (80 > years old) individuals of both genders know fewer number of plants and uses than those of middle age (32–65 years old), this agrees with the results found in other countries of [9,27,28]. Test of Kolmogorov-Smironov for both, women (number of plants: *K-S* = 0.55, *d.f* = 55, *P* > 0.20; uses: *K-S* = 0.098, *d.f.* = 55, *P* = 0.200) and men (number of plants: *K-S* = 0.104, *d.f.* = 55, *P >* 0.20; uses: *K-S* = 0.106, *d.f.* = 55, *P >* 0.185) and Levene test (number of plants: *F* = 1.049; number of uses; *F* = 1.391) shown normality and homogeneity of variance respectively. The *t* Test showed that women know more plants (*t =* 2.93*, d.f. =* 108*, p >* 0.007) and more uses (*t =* 2.703*, d.f. =* 108*, p >* 0.008) than men in Rayones. There was a very low non-significant correlation between age of the people and the number of species known (*r* = -0.087, *n* = 110, *Pr* > 0.366) (Figure 2), but, there was a very low significant negative correlation between age of the individuals and the number of uses (*r* = -0.239, *n* = 110, *Pr* > 0.012) (Figure 3).

### Diversity of useful plants

Two hundred and fifty two species, 228 genera and 91 families of vascular plants and one family, one genus and one species of fungus were recorded (Additional file 1). The Table 1 shows the different groups of plants and fungi recorded as well as quantitatively the most diverse families regarding genera and species, main growth forms, the origin and the provenance of these. Dicotyledonous represent almost 90% of useful flora. Fourteen of the families include 42% of the genera and 54% of the species. Wild (60%), autochthonous (76%), herbaceous (52%) and shrubby (25%) species are the most used. The most important families with useful wild species are: Asteraceae, Fabaceae, Cactaceae, Euphorbiaceae and Agavaceae, while the most important families with useful cultivated ones are: Poaceae, Rosaceae, Rutaceae and Solanaceae. The wild genera with the highest number of useful species are *Agave* (5), *Opuntia* (4), *Euphorbia* (3) and *Acacia* (3), while cultivated genera with the higher number of species are: *Citrus* (4), *Prunus* (3), *Capsicum* (2), *Cucurbita* (2), and *Allium* (2).

**Table 1:** Main groups of useful vascular plants (and fungus), families with the highest number of genera and useful species, and the origin and provenance of them recorded in Rayones, Nuevo León, México

Main groups of useful plants (and fungus) (families, genera, and species respectively)	Dicots. (64, 190, 208); monocots. (20, 35, 41); conifers (3, 3, 3); ferns and allies (3, 3, 3,); fungi (1, 1, 1).
Families with the highest number of genera and species respectively	Asteraceae (19, 22), Fabaceae (11, 16), Lamiaceae (11, 11), Rosaceae (10, 14), Poaceae (8, 8), Cactaceae (7, 11), Euphorbiaceae (7, 9), Solanaceae (6, 10), Rutaceae (5, 8), Verbenaceae (5, 6), Cucurbitaceae (4, 6), Oleaceae (4, 5), Apiaceae (4, 4), Boraginaceae (4, 4), and Agavaceae (3, 7),
Main growth forms	Herbaceous (128), shrubs (63), trees (30), succulent (28), parasites (3)
Origin	Wild (157), cultivated (95)
Provenance	Autouchthonous (193), introduced (59)

### Number of uses and ways in which the plants are used

**Figure 4:**
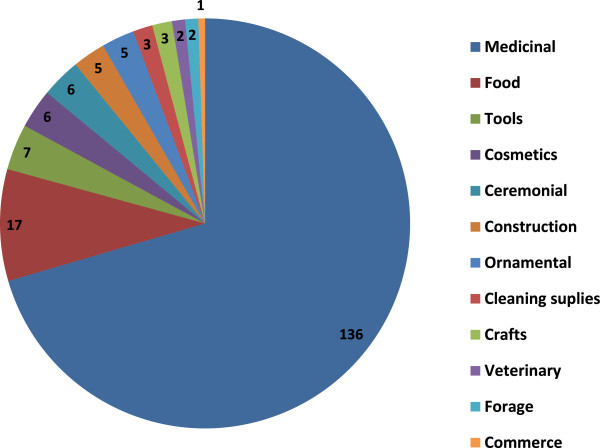
Main categories of use of plants and number of species per category in Rayones, Nuevo, León, México.

**Figure 5:**
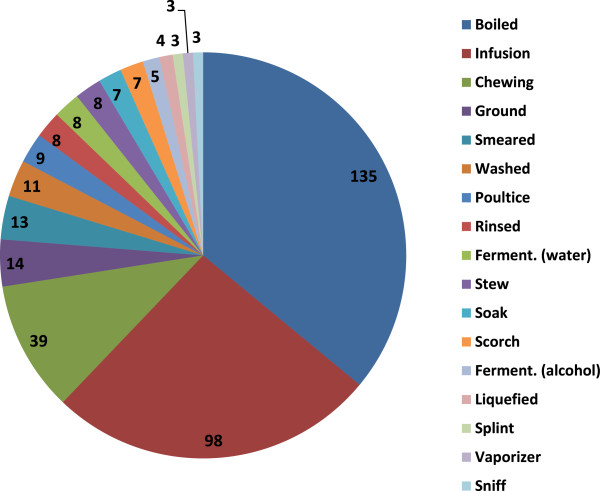
Main categories and number of species per category in which plants are used in Rayones, Nuevo, León, México.

We recorded 193 different uses in 12 different categories. Medicinal use is by far the most important, it represents 71% of the total uses, followed by food (9%) and utensils and tool (4%) uses (Figure 4). We recorded 17 different categories in which plants are used, the five more important ways of uses are: boiled (135 species), infusion (98 species), chewing (38 species), milled (13 species), and smeared (12 species) (Figure 5).

### Parts of plants most commonly used

**Figure 6:**
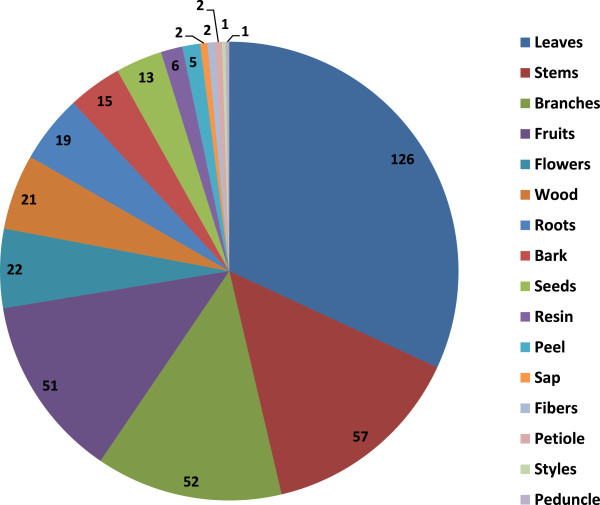
Morphological constituents of the plant most commonly used in Rayones, Nuevo León, México.

People from Rayones utilize almost all parts of the plant, however, the leaves, are by far, the most commonly used part, we recorded 126 species which the leaves are used in different ways, followed by the logs (stems) (57 species), branches (52) and fruits (51) (Figure 6).

### Medicinal plants

**Figure 7:**
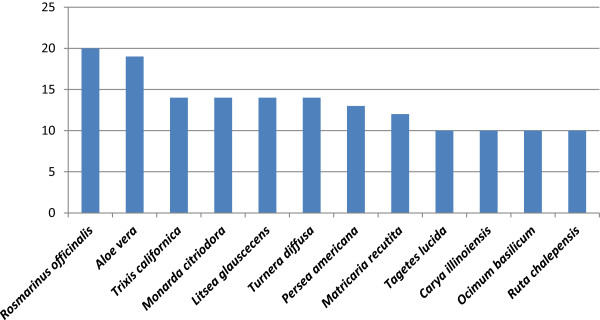
Medicinal plants with 10 or more uses in Rayones, Nuevo León, Mexico.

**Figure 8:**
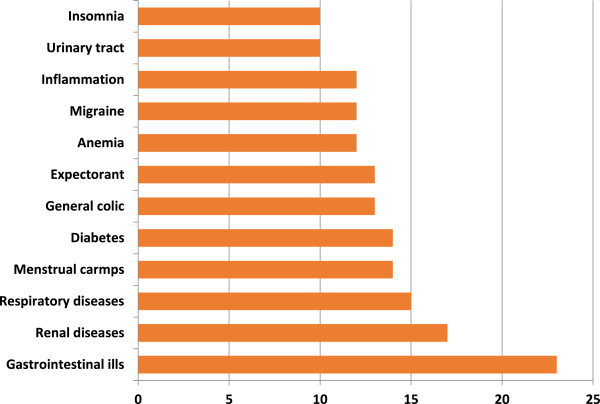
Main illness recorded and number of medicinal species used to heal them in Rayones, Nuevo León, Mexico.

Of all the species recorded, 160 of them are used as medicinal, they includes 64 families and 139 genera. The families with the highest number of medicinal species are Asteraceae (19 species), Lamiaceae (11), Cactaceae (9), Euphorbiaceae (9), Rutaceae (7), Agavaceae (6), Fabaceae (6) and Rosaceae (6). The genera with the higher number of medicinal species are: *Agave* (5), *Acacia* (3), *Citrus* (3), *Euphorbia* (3), *Opuntia* (3), *Allium* (2), *Artemisia* (2), *Commelina* (2), *Cucurbita* (2) and *Dyssodia* (2). Twelve of the species has at least 10 different medicinal uses (Figure 7). According to the information recorded, several species are used to heal different ailments, such as gastrointestinal ills, renal diseases, respiratory diseases, menstrual cramps, and diabetes (Figure 8).

### Food plants

Food plants are used in 11 different ways such as edible fruits (40 species), condiment (20), tea (16), green salad (13), food supplement (13) and confectionery (11). The families most commonly used were Rosaceae (11 species), Lamiaceae (10), Cactaceae (6), Rutaceae (6), Asteraceae (4) and Solanaceae (4). The genera with the higher number of food plants are *Agave* (5), *Opuntia* (4), *Citrus* (4), *Prunus* (4) and *Cucurbita* (3). The species with the highest number of different food uses are *Persea americana* var. *drymifolia* (5), *Citrus limon*, (4), *Cucurbita moschata* (4), *Tagetes lucida* (4), and *Agave americana* var. *americana* (4).

### Ritual or ceremonials

At least, 24 genera (11 introduced and 13 native) and 26 different species (11 introduced and 15 native) are used as part of religious ceremonies or spiritual rituals. According to the information recorded, the most common uses and the number of species respectively used are: to frighten or keep away (12), ceremonial (11), mystical (11), purify the soul (10), ill will (8) and good look (7). The five most commonly plants used in the ritual are: *Ruta chelepensis*, *Aloe vera*, *Salvia microphylla, Brahea dulcis* and *Mentha piperita*.

### Wood and construction

Forty species in the area are used for purposes related to construction and wood. Most of species used are shrubs (20) and trees (14). Fourteen of the species has at least 3 or more different uses. Four of them has at least four different uses (*Acacia berlandieri, Eysenhardtia texana, Helietta parvifolia,* and *Prosopis glandulosa* var. *torreyana*), two of them has at least five (*Havardia pallens* and *Juglans major*), and only one has at least seven different uses (*Carya illinoiensis*). Nineteen species are used for fences, 16 are used for home construction, 14 for firewood, 11 for household woods, and 11 for wood in several ways. Legumes are the most important species for wood and construction activities.

### Ornamental

Wild and cultivated species are commonly given an ornamental use. According to their characteristics, five different categories were mentioned by respondents, a) aromatic plants, b) flowers, c) leaves, d) aesthetic and e) shade. In total, 75 species are given an ornamental use, especially for their general beauty (26), flowers (26), and for shade (11). Only eight plants were selected for their leaves or for their scent (2). Nine species has, at least, two different uses as ornamental, beauty-flowers, beauty-leaves, beauty-shade, beauty-scent, flower-leaves, etc.: *Bauhinia purpurea, Bougainvillea spectabilis, Chilopsis linearis, Echinocereus platyacanthus, Justicia spicigera, Marginatocereus marginatus, Mentha piperita, Pelargonium hortorum,* and *Washingtonia filifera*.

### Cosmetics

Twenty-three different species are used in several ways to make soap, shampoo, foot powder, lice shampoo, hair dye, hair health products, and firming skin tonics. Plant parts of *Equisetum leavigatum*, *Aloe vera* and *Rosmarinus officinalis* (leaves), *Jatropha dioica, Loeselia mexicana, Simmondsia chinensis, Agave lecheguilla,* and *Aloe vera* (root), *Juglans major* (fruit peel) and *Cucurbita foetidissima* are used to make shampoo. Roots of *Acacia rigidula*, *Jatropha dioica,* and *Glandularia bipinnatifida*, fruits of *Morus celtidifolia* and leaves of *Fraxinus cuspidata, Tragia ramosa* and *Salix humboldtiana* are used to manufacture products for hair care. Four species are commonly used to make lice shampoo (*Chenopodium ambrosioides, Hechtia scariosa, Litsea glauscecens,* and *Azadirachta indica*). Milled dried leaves of *Trixis californica* var. *californica, Larrea tridentata* and *Matricaria recutita* are commonly used to make foot powder*.* The fruits of *Cucurbita foetidissima*, a very common weed plant are also used to make soap.

### Other uses

Several particular species such as *Agave americana* var. *americana* (sap and pulp) *Opuntia imbricata* (sap and pulp) and *Trixis californica* (leaves) are used to heal broken bones legs in animals by splints; the “piloncillo” (the name given in Mexico to solid unrefined cane sugar) is frequently used to make sweets; milled dried leaves of *Prunus persica*, mixed with fat and clay are commonly used to remove worms from animal skins; dried fruits of *Lagenaria siceraria*, open at both ends, especially those with slim and elongated apex are used as devices to extract sap from maguey by suction; dried and milled leaves of *Solanum douglasii* are used as rat poison; branches and leaves boiled of *Phaseolus vulgaris* and *Phoradendron villosum* mixed with salt are given to the goats to shed the remnants of the placenta; mature, but still fleshy fruits from *Cucurbita foetidissima* are used by women to extract the placenta after birth; the *Datura stramonium* seeds added to the food are used to allegedly fall in love with a person.

## Conclusions

### Knowledge of plants and uses

As far as we know, the documented information about plants and their uses in both, central and southern region of the state of Nuevo León [11,12], women have a greater knowledge about plants and their uses than men. Women from Rayones are not the exception. On average, women know 14 more plant species and 12 more plant uses than men. Women from Rayones, besides their common activities (child care, cook), they habituate to collect plants in the field, especially medicinal ones to sell them in order to help in the family economy, similar knowledge has been reported for women in Zimbabwe and Mexico [6], Nicaragua [15], Mexico [16,17], and Brazil [18], much of this knowledge is related to plants used for female conditions such as pregnancy, childbirth and menstrual problems [15], and for general use as carers of plants in transformed spaces and in home gardens [6]. In average, women and men of middle age from Rayones know a bigger number of plants and their uses than younger and older residents. Younger residents were the sector with less knowledge about plants, this is, in part because young people have access to new technologies and modernization which sets them apart from traditional knowledge, similar causes of this loss of traditional botanical knowledge has been recorded in Turkey [28]. Unexpectedly for us, in Rayones, older individuals of both genders mentioned fewer plants and uses those middle-aged. Several of the older residents interviewed mentioned that 10–20 years before, they remembered more species and more uses than in current days, it agrees with results recorded in Brazil [8], Ethiopia [9] and South Africa [27]. Our interpretation about why women know more plants and uses than men, is essentialy because women know quantitatively, more medicinal plants and their uses than men, and since that use is the most relevant one for Rayones residents, the amount of medicinal plants and uses known far outweight the rest of the other species and their uses, including such as timber and food, widely known by men. Many of the women interviewed assume, at home, the role of health care for the family as well as their close relatives, especially the elderly. As such, they strive to know what type of plants can be used to cure certain ailments and often, they use them before, during and even after consultation with a doctor. With regard to age-number of plants and age-number of uses relatonships and according to our interviews, younger individuals are those who know less plant and uses. This age-sector know the most common edible cultivated plants such as corn, oat, calabaza, peach, etc., and, also, they know, but few, the most common wild plants such as mezquite, gobernadora, tenaza, huizache, nopal, chochas, etc., nevertheless, in many cases, they do not know anything about their uses, and when asked about medicinal plants, their ignorance is even greater. Most of the respondents when asked about certain type of medicinal plant and its use, their response was “I do not know about it, but my parents do”, when we asked them the cause of the ignorance, most replied that they were not interested as much as their parents, because they preferred go to doctor or take pills intead of drinkig an infusion of “dried leaves”. Based on the responses, we infer that new generations are becoming less interested in learning and using regional cualtivated or wild plants, since the collection of plants, drying and use them is perceived as an outmoded practice, dated, and only used by old people. Forgetfulness, disuse and memory loss are the main causes of the low number of plants known by older people. Almost half of the respondents did mention that they had troubles remembering several species and their uses; they mention that disuse of many species over time has influenced their forgetfulness. As far as we know, middle-aged and older residents will continue using the different plants for their own purposes, but, is unlikely that younger residents continue this tradition. In the context of the Mexican Ethnobiology, this regional study of useful plants provides a broader understanding of the diversity of uses existing in northeastern region, which helps to know similarities and differences from other regions of Mexico, but also, provides information about its particular flora and their uses, absent in other areas.

### Diversity

Surprisingly for us was to find that in Rayones, people knew more plants and more uses than in the central part (which includes six municipalities) [11] and the southern part of the state of Nuevo León (which includes three municipalities) [12]. This is because both, men and women collected plants in field, and the rich plant diversity found in this area, since two important ecosystems, Sierra Madre Oriental (temperate) and the Chihuahuan Desert (arid) converge in this area. Interviewees mentioned that they collect and exchange plants with each other, and also, they exchange knowledge of plants and how they should be used. In Rayones as in the central and the southern region of the state of Nuevo Leon, the families Astraceae, Fabaceae, Cactaceae, Euphorbiaceae and Agavaceae are also the most diversified in wild species. The most diversified families with cultivated species are Rosaceae (mainly trees), Poaceae, Solanaceae, Rutaceae, and Cucurbitaceae. In Rayones, most of plants known and used by residents are herbaceous, wild and autochthonous species. The rich species diversity of wild genera such as *Opuntia*, *Agave*, *Euphorbia* and *Acacia*, as well as cultivated ones such as *Citrus*, *Capsicum*, *Prunus*, *Cucurbita,* and *Allium*, allow residents to use them in many different purposes. Several families such as Poaceae, Solanaceae, Cactaceae, Rosaceae, Fabaceae, Rutaceae, and Cucurbitaceae in Rayones as in other parts of México stand out as the most useful genera and species with different purposes in northeastern and southern of México [29-36].

### Uses, way of use and plant parts used

Quantitatively, the medicinal is the most important use of plants in Rayones, 70% of the different uses concern this, followed in importance by food and timber species. Boiled (36% of the species) and infusion (26%) are the main ways of use. Despite of all plant parts are commonly used for diverse purposes, several plant parts such as leaves (47%), branches (22%), and fruits (20%) are most commonly used. In Rayones as in parts of Oaxaca [37], leaves, stems, flowers, sap, roots, and fruits are the most common plant parts used; both areas share several commonly genera used such as *Aloe*, *Apium*, *Carya*, *Citrus*, *Lippia*, *Ocimum*, *Persea*, *Rosmarinus*, *Ruta*, *Solanum*, and *Tanacetum*.

### Medicinal plants

Sixty seven percent of the species known in Rayones are used as medicinal in 16 different ways mostly boiled or as infusion. The most common practice is to prepare a tea by boiling a certain amount dried leaves or as an infusion with dried leaves to heal most ailments. Rayones has a higher percentage of plants used as medicinal than in the central [11] or southern area [12] of the state of Nuevo León, it could, in part because Rayones is smaller town and it has fewer medical services than other parts of central and southern state of Nuevo León, and also, because they are in close contact with the plants they collect, in addition to being less walking distance to obtain them in the field. Most houses were the interviews were carried out cultivated several common species, such as *Opuntia ficus-indica, Agave americana, Citrus* spp., *Matricaria recutita, Rosmarinus officinalis, Yucca* spp., *Punica granatum, Ficus carica, Persea americana, Carya illinoiensis, Capsicum annum, Prunus persica,* and *Zea mays,* most of them are commonly used as medicinal or food in many different ways. Only two people in Rayones are engaged in the wholesaling of medicinal plants. They manufacture their own products such as foot powder, shampoo, lice shampoo. Most of the manufactured products are mixed with glycerin to preserve them for a longer time; otherwise, these are only macerated, milled or broken in smaller parts to sell. Two microendemic and endangered species [38,39], *Aztekium ritteri* (known regionally as *peyote* or *false peyote*) and *Ariocarpus scaphirostris* (known regionally as *cockscomb* for its peculiar pointy mamilas) are sold in small markets, since people in the region know that both species are endangered, even so these cacti are sold illegally in the village by local or foreign collectors (outside of the village). Both species are sold as substitute for peyote (*Lophophora williamsii*), and are commonly used in alcohol as poultice against arthritis and fatigue.

### Food plants

Several introduced tree species are very common in most gardens of Rayones; they are planted for self-consumption or for sale, these include peaches, plums, apricots, pomegranate and fig. Most of fruits are eaten raw, or sold as canned products. Some small areas (3–4 ha surface) outside the Rayones village, still grow sugar cane (*Saccharum officinarum*), people produce mead (regionally called *aguamiel*) and piloncillo (solid unrefined cane sugar), both products are mainly sold regionally. A common practice of most residents is to cultivate *nopales* (*Opuntia* spp.) in their gardens, this autochthonous genus has a relevant importance in the regional diet, since it is a perennial plant, it does not need special care to grow, its fleshy edible stalks and fruits are present most of the year, it is easy to reproduce (vegetatively), such virtues make *nopal* one of the most frequent food in the regional diet, and cooked in multiple recipes. Not as common as the nopales, but also frequent, the *chochas* or called also *flor de palma* (open flower but even without being pollinated) of several species of the genus *Yucca* are highly appreciated in the regional cuisine. In the flowering season (March to April), people gather complete inflorescences and sell them (reaching prices about $30.00 to $50.00 pesos, about 4–5 dollars each). Once pollinated, the flowers take a bitter taste and the prices decrease significantly and often the inflorescences are no sold. This seasonal food is a tradition in many places in northeastern Mexico. Another edible product with high demands is the *aguamiel* (sap) of the maguey (*Agave* spp.). Rayones’s residents recommend drinking this sweet sap instead of sugar (after boiling it) to diabetics. People do not recommend drinking the sap uncooked, as it cause diarrhea. Two and a half litters of sap are sold in $30.00-$50.00 pesos (about 3–3.5 dollars). Several autochthonous wild or cultivated species with edible fruits such as *Crateagus greggiana* var. *greggiana, Cucurbita moschata, C. ficifolia*, *Persea americana* var. *drymifolia*, *Physalis philadelphica*, *Capsicum annum*, and other species with edible leaves such as *Tagetes lucida, Monarda citriodora* var. *austromontana*, *Litsea glauscecens*, *Lippia graveolens*, *Hedeoma drummondii*, *Amaranthus palmeri*, *Croton suaveolens, Portulaca mundula*, are frequently gathered to prepare salads, beverages or used as condiments. Roasted and raw roots of several *Hechtia* species are not commonly used for food in Rayones, but for the making of shampoo, while in northwestern Mexico, these roots are commonly roasted for food [40]. *Begonia* is a common plant cultivated in gardens as an ornamental in Nuevo León, however in Puebla, the petioles of at least six different species of *Begonia* are commonly used as food [41]. Other species such as *Echinocactus platyacanthus* (candy barrel cactus) widely distributed in the Chihuahua Desert are used in different states such as Hidalgo, Querétaro, and Oaxaca to make candy or used as forage for cattle [42], in Nuevo León this species is used in a similar way. Several edible Cucurbitaceae found in Nuevo León [11] are also widely used as food or soap substitutes, such as *Cucumis anguria* (fruits), *Cucurbita foetidissima* (fruits). The quelites (*Amaranthus* spp.) are a weed species in Nuevo León, however, its leaves and inflorescences are widely used as food [43], in Rayones, *Amaranthus palmeri* is one of the most common foods along with nopales (*Opuntia* spp.).

### Wood, fuel, tools and construction

**Figure 9:**
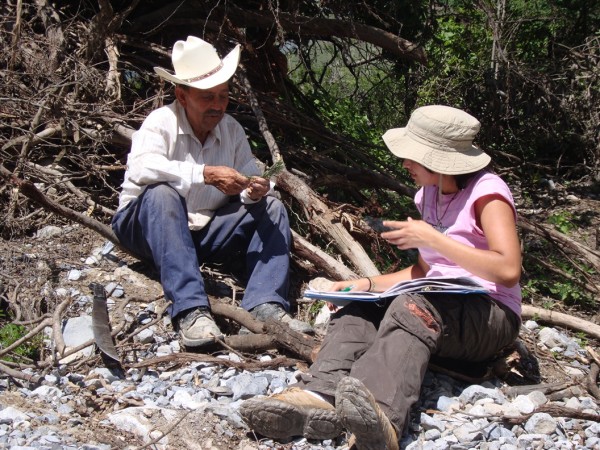
One of the interviewees in Rayones, Nuevo León while cutting wood in the field.

Almost 18% of the flora is used for wood and construction purposes. Because of their size, thickness and hardness, *Prosopis glandulosa* var *torreyana*, and *Helietta parvifolia*, are by far, the most frequently used species for house construction and fences; both of them have a strong wood, suitable for these activities. According to information gathered from the residents (men) (Figure 9), fences made from *Helietta parvifolia* logs last for about 30 years. Other species such as *Acacia berlandieri*, *A. farnesiana*, *Havardia pallens* are commonly used in the construction of houses, roofs, fences, but also, they are an excellent source for charcoal, part of tools (hammer handle, handle hoes, sickle handle) and firewood. The wood is not marketed locally; they use them to meet their own needs. Besides these uses, some species are multipurpose such as *Carya illinoiensis* (fruits, wood, fuel, construction and shampoo), *Eysenhardtia texana* (forage, medicinal and fuel)*, Juglans major* (fruits, wood, fuel, construction and shampoo). Besides being used for food, mezquite and alien species are used in similar way by the aborigines in northwestern Mexico and southwestern USA [44]. In Tamaulipas, *Prosopis glandulosa, Acacia farnesiana, Havardia pallens, Helietta parvifolia*[29] are commonly used for making fences or used as fuel (*Ebenopsis ebano*), the same species and the same uses are practiced by residents of Rayones. Knowledge of timber species used for construction, household’s goods, tools and crafts is greater in men than in women in the study area, similar results were recorded in Zimbabwe [6], southern [16] and north of México [17].

### Ornamental and cosmetics

The physiognomic beauty and showy flowers are the main characteristics selected by residents to cultivate ornamental plants. Because of their easy availability in the field and because of the perennial habit of the species, the Cacatceae (*Echinocactus, Opunita, Marginatocerus*) and Agavaceae (*Agava* and *Yucca*) are the most conspicuous plants in most gardens, not only for ornamental purposes, but also, for living fences, food, sap, medicinal uses, etc. Almost 30% of the total flora recorded is given an ornamental use. Most gardens in the study area cultivate herbaceous, shrubs and trees, and are commonly used as food, condiments and medicinal purposes such as *Bougainvillea spectabilis, Chilopsis linearis, Echinocereus platyacanthus, Justicia spicigera, Marginatocereus marginatus, Mentha piperita, Pelargonium hortorum,* and *Washingtonia filifera*, among others. Several different cosmetics, firming skin lotions and tonics and health hair products uses recorded in Rayones have not been reported for other areas in the state of Nuevo León, especially those species used for make shampoo or used as shampoo such as *Equisetum leavigatum, Rosmarinus officinalis*, *Jatropha dioica, Loeselia mexicana, Simmondsia chinensis, Agave lecheguilla, Juglans major* and *Cucurbita foetidissima*. As far as we know [11,12], no other areas in the State of Nuevo Leon use wild autochthonous plants such as *Chenopodium ambrosioides, Hechtia scariosa,* and *Litsea glauscecens,* and also one introduced species (*Azadirachta indica*) to make shampoo for lice control. Rayones is the one of the few municipalities where products manufactured from wild and cultivated plants are marketed on a small scale.

### Uses in Rayones and other areas of Nuevo León and north of Mexico

One of the first plant collections of useful plant in northeastern Mexico (Coahuila, San Luis Potosí, and Tamaulipas) was carried out by Palmer [45], this set of plants is currently stored at the Peabody Museum (Harvard University), and several of the plants mentioned are used in Rayones in similar way. As in other parts of Nuevo León and north of Mexico, the peyote and its psychoactive compounds are used to heal arthritis, but also for its hallucinogenic properties [46,47]. *Echinocactus platyacanthus* is commonly used to feed goats and to make candies, the populations of this species and the ones of *Ferocactus histrix* are severely impacted for these uses [48], currently, and both of them are endangered species in México. The quelites (*Amaranthus*) are widely used in Mexico as food [49], the quelites are a common food in the diet of the Rayones residents. The lechuguilla production [50], wood use [51], amole root used as shampoo[52], ixtle fiber extraction [53], consumption of wild beans [54], laurel oils uses [55] recorded in the north, central and south of Mexico are also common practices of routine use in Rayones; all of them are similarly used in Rayones for same purposes. People still produce lechuguilla fibers, however, this practice is almost vanished because of the low prices of the fibers. Several roots (*Agave* and *Opuntia*) are commonly used for make soap, food or for medicinal use in a similar way to the *chicana* (*Hechtia*) roots [40], and other species of *Agave* from northwestern Mexico [56]. In Tamaulipas, 610 useful species were recorded, including 334 medicinal and 154 timber species [57], almost 30% of them are found in Rayones. For the arid areas of Tamaulipas, 53 medicinal species have been recorded [58], many of these species are present and used in Rayones in a similar way, especially the herbaceous and shrub species. In Coahuila, the ethnic Kikapoo uses at least 150 useful plant species, 26 of them are widely used as medicinal [59], almost 12% of them are commonly used in Rayones. In northwestern Mexico, the Raramuri ethnia uses dozens of medicinal plants, and has been compared to those found in city markets [60], some of them are used as a poisonous plants [61], only one species (*Solanum douglasii*) is used as a poisonous plant in Rayones. In Chihuahua it is known that many Raramuri kids know up to 40 different useful species [62], greater amount than that in younger individuals interviewed in Rayones. Raramuri women know at least 356 different useful species, highlighting those medicinal, forage, food, fuel and construction [17]. In Sonora, where Pima and Yaqui ethnicity persist, still uses several plants as living fences [63] and many species are used as food [64], several of these genera are also used in Rayones for the same purpose. Of the nearly seventy medicinal plants recorded on the market of Monterrey [65], almost 60% of them are used in Rayones in similar way, especially the medicinal species. Almost 70% of plants recorded in Rayones are used also in similar way in the Cumbres de Monterrey National Park [11], and almost 80% of the useful flora recorded in Rayones was recorded in the southern area of Nuevo León, México [12].

## Consent

Written informed consent was obtained from the patient for the publication of this report and any accompanying images.

## Competing interests

The authors declare that they have no competing interests.

## Authors’ contributions

The field work and database confection was carried out by EEC, MGL, BSM, MRS, and JVQ, data analysis was carried out by EEC, HGR, CCA, DGU, ICS and ACP. Manuscript preparation was conducted by all authors. All authors read and approved the final manuscript.

## Supplementary Material

Additional file 1 **List of useful plants used in the Municipality of Rayones, Nuevo León, México.** Number after plant author MG (Miriam Garza, number of collection). Click here for file
