# Supplementary material for: Ethnobotany in Rayones, Nuevo León, México
Source: J Ethnobiol Ethnomed. 2014 Sep 1;10:62. doi: 10.1186/1746-4269-10-62 (PMC4237796; doi:10.1186/1746-4269-10-62)
Supplement: Additional file 1 — List of useful plants used in the Municipality of Rayones, Nuevo León, México. Number after plant author MG (Miriam Garza, number of collection). [file 1746-4269-10-62-S1.pdf]

Additional file 1: List of useful plants used in the Municipality of Rayones, Nuevo León, México. Number after plant author MG (Miriam Garza, number of collection).

**Scientific name**	**Common name**	**Uses**	**Part used**	**Method of use**
ACANTHACEAE				
Justicia spicigera Schltdl**.,** MG 255	Tintoreta	Ornamental	Complete plant	Planted in gardens
AGAVACEAE				
Agave americana L. var *americana*, MG 327	Maguey	Urinary system	Sap	Boiled, drink
		Food	Flowers	Cooked
			Sap	Mezcal (fermented)
		Living fences	Plant	Planted in the gardens
		Ornamental	Plant	Planted in the gardens
		Forage	Leaves	Raw
		Veterinary	Pulp leaves	Splinting borken legs
Agave *bracteosa* S.Watson ex Engelm., MG 349	Amole de castilla	Inflammation	Root	Boiled, as poultice, applying directly on the affected area
		External schoks	Root	Mixed with canela and *Acacia farnesiana* bark chips
Agave lecheguilla Trel., MG 328	Lechuguilla	Bruises	Root (pulp)	Boiled, drink the solution
		Inlammation	Root (pulp)	Boiled, as poultice, applying directly on the affected area
		Internal wounds	Root (pulp)	Boiled, drink the solution
		Purify blood	Root (pulp)	Boiled, drink the solution
		Depression	Root (pulp)	Boiled, drink the solution
		Stomach ache	Root (pulp)	Boiled, drink the

				solution
		Alopecia	Root (pulp)	Boiled, spread the solution on the head like shampoo
		Forage	Root; flowers	Ground, raw; raw
		Food	Flowers	Cooked, with eggs and bacon
		Fibers	Leaves	Scraped into fibers, to make laves
		Dandruff	Root (pulp)	Dried, ground, then boiled and spread over the head
Agave lophantha Schiede ex Kunth, MG 326	Lechuguilla de castilla	Pains	Root	Boiled, drink as tea
Agave scabra Ortega, MG 471	Maguey or cuajo rayado	Prevention of heart attack	Stem pulp	Boiled, drink the solution
Brahea dulcis (Kunth) Mart., MG 348	Palmito	Fibers	Young leaves	Woven fibers to make chair and benches
		Ceremonial	Complete leaves	Part of the bouquets
Polianthes tuberosa L., MG 350	Nardo	Ornamental	Complete plant	Cultivated in the gardens
Yucca filifera Chabaud, MG 413	Palma datilera	Food	Fruits	Raw, sweet flavor
		Food	Flowers	Called “chochas”, cooked, very popular food
		Living fences	Complete plant	Planted in gardens
		Ornamental	Complete plant	Planted in gardens
AMARANTHACEAE				
Amaranthus palmeri S. Watson, MG 256	Quelite	Anemia	Leaves and inflorescences	Boiled, drink the solution
		Food	Leaves	Cooked
		Forage	Complete plant	Dried, raw
AMARYLLIDACEAE				
Eucharis grandiflora Planch. & Linden, MG		Ornamental	Complete plant	Planted in gardens

325				
		Hepatitis	Leaves	Boiled, take a bath with the solution
ANACARDIACEAE				
Rhus virens Lindh. ex A. Gray, MG 257	Lantrisco	Diabetes	Leaves and branches	Boiled, drink as tea
		Cholesterol	Leaves and branches	Boiled, drink as tea
		Menstrual cramps	Leaves and branches	Boiled, drink as tea
		Revitalizing	Bark	Boiled, drink as tea
Schinus molle L., MG 414	Pirul	Cataracts		Ground applied directly, applying directly into eyes
		Ceremonial	Leaves	Purify the soul
		Ornamental	Complete plant	Planted in the gardens
APIACEAE				
Apium graveolens L., MG 351	Apio	Food and condiment	Leaves	Raw or cooked
Coriandrum sativum L., MG 352	Cilantro	Condiment	Leaves	Raw or cooked
Eryngium heterophyllum Engelm., MG 448	Hierba del sapo	Cholestreol and diabetes control	Branches and leaves	Boiled, drink as tea
Foeniculum vulgare Mill., MG 353	Hinojo	Intestinal colic	Leaves	Boiled, drink the solution
		Colic cramps	Leaves	Boiled, mixed with canela bark, drink as tea
		Promote milk production in women	Leaves	
Petroselinum sativum Hoffm., MG 347	Perejil	Stimulate appetite	Leaves	Raw, in salads
APOCYNACEAE				
Nerium oleander L., MG 470	Laurel rosa or laurel blanco	Ornamental	Complete plant	Planted in gardens (leaves highly poison if eaten)
ARACEAE				
Syngonium podophyllum Schott, MG 449	Julieta	Ornamental	Complete plant	Planted in gardens

Xanthosoma sagittifolium (L.) Schott & Endl., MG 415	Lampazo or cabezona	Ornamental	Complete plant	Planted in gardens
Zantedeschia aethiopica (L.) Spreng., MG 469	Alcatraz	Ornamental	Complete plant	Planted in the gardens
ARECACEAE				
Washingtonia filifera (Linden ex André) H.Wendl. ex de Bary, MG 446	Palma	Ornamental	Complete plant	Planted in gardens
		Forage	Flowers	Raw, for goats
		Food	Flowers	Cooked
ASCLEPIADACEAE				
Asclepias oenotheroides Schltdl. & Cham., MG 450	Hierba del cizote	Cizotes (fungal diseases)	Sap milk	Applied directly on the affected part
ASPARAGACEAE				
Sansevieria thyrsiflora (Petagna) Thunb., MG 282	Huaco, guaco or lengua de suegra (mother in law tongue)	Bruises	Leaves	Ground, applied directly in the affected part
		Insect bite	Leaves and root	Ground, applied directly in the affected part
		Inflammation	Leaves and root	Ground, applied directly in the affected part
		Gastritis	Root	Fermented, drink the solution
		Ceremonial	Complete plant	Bouquets
		Ornamental	Complete plant	Planted in gardens
ASPHODELACEAE				
Kalanchoe daigremontiana Raym.-Hamet & H. Perrier, MG 354	Oreja de burro	High fever	Leaves	Ground, strain the pulp, applied as poultice, and also, drink the remainder
Aloe vera (L.) Burm.f., MG 357	Sábila	Acne	Leaves	Ground, as poultice in the affected area

		Cicatrizant	Leaves	Ground, as poultice in the affected area
		Skin diseases	Leaves	Ground, as poultice in the affected area
		Insect bites	Leaves	Ground, as poultice in the affected area
		Remove spines from the skin	Leaves	Ground, as poultice in the affected area
		Inflammation	Leaves	Ground, toasted, as poultice in the affected area
		Skin burn	Leaves	Ground, toasted, as poultice in the affected area
		Lose weight	Leaves	Ground, as poultice in the affected area
		Diabetes	Leaves	Liquefied, drink on an empty stomach
		Cough	Leaves	Liquefied, drink
		Gastritis	Leaves	Liquefied, drink on an empty stomach
		Cancer	Leaves	Liquefied, drink
		Pain of angina	Root	Ground, drink as tea
		Backache	Leaves	Ground, as poultice in the affected area
		Ceremonial	Complete plant	Bouquets
		Wounds	Leaves	Ground, as poultice in the affected area
		Intestinal colic	Leaves	Ground, raw, eat
		Bracing	Leaves	Liquefied, raw, drink
		Antibiotic	Leaves and root	Liquefied, raw, drink

Asphodelus fistulosus L., MG 356	Cebollín or cebolleta	Varices	Leaves and bulbs	Ground, spread the pulp in the affected area
ASTERACEAE				
Achillaea millefollium L., MG 346	Real de oro, milenrama	Intestinal colic	Branches and leaves	Boiled, drink as tea
		Menstrual cramps	Branches and leaves	Boiled, drink as tea
		Strengthen gums	Branches and leaves	Boiled, swish around the mouth, drink as tea
		Ceremonial	Complete plant	Dried, passed over the body (frightened people)
Artemisia absinthium L., MG 355	Ajenjo	Stomach pain	Leaves	Boiled, drink as tea
Artemisia ludoviciana Nutt., MG 473	Estafiate	Intestinal colic	Leaves	Boiled, drink as tea
		Diarrhea	Leaves and inflorescences	Boiled, drink as tea
		Intesitinal parasites		Boiled, mixed with leaves of *Matricaria recutita*, *Prunus persica* and *Mentha piperita*, drink the solution
		Vomit	Branches	Boiled, drink as tea
		Condiment	Leaves	Cooked with the meal
Baccharis salicifolia (Ruiz & Pav.) Pers., MG 416	Jara o jarilla	Stomach ache	Leaves	Chewed
		Household woods	Dried stems and branches	To make brooms
		Smelly feet	Leaves	Raw, put them inside shoes
Chrysactinia mexicana A. Gray, MG 345	Hierba de San Nicolás	Aphrodisiac	Branches	Boile, drink as tea, one cup for nine consecutive days
		Fertility (mean	Branches	Boiled, drink as

		and women)		tea
		Menstrual cramps	Leaves and branches	Boiled, drink as tea
Dahlia pinnata Cav., MG 474	Dalia	Ornamental	Complete plant	Planted in gardens
Dyssodia acerosa DC., MG 475	Hierba del zorrilo	Cough	Leaves	Boiled, six to seven leaves, drink as tea
Dyssodia setifolia (Lag.) B. L. Rob., MG 476	Parraleña	Diarrhea	Leaves	Boiled, drink as tea
Echinacea angustifolia DC., MG 344	Quina, equina, equinácea	Anemia	Leaves	Boiled, drink as tea
		Antibiotic	Leaves	Boiled, drink as tea
		Flu	Leaves	Boiled, mixed with *Melissa officinalis* and *Cymbopogon citratus* leaves, drink as tea
		Beverage	Leaves	Boiled, drink as tea
		Ornamental	Complete plant	Planted in gardens
Flourensia cernua DC., MG 371	Hojasé	Intestinal colic	Leaves	Boiled, six to seven leaves, drink as tea
		Colic cramps	Leaves	Boiled, six to seven leaves, drink as tea
		Constipation	Leaves	Ground and toasted, six to seven leaves for two cups, mixed with honey bee and avocado leaves, drink as tea or, also, add a tablespoon of baking soda, drink it during three days along with a roasted chicken gizzard

		Diarrhea	Leaves	Boiled, six to seven leaves, drink as tea
		Stomach infection	Leaves	Boiled, six to seven leaves, drink as tea
Helianthus annuus L., MG 477	Girasol	Forage	Complete dry plant	Raw
		Ornamental	Complete plant	Planted in gardens
Gnaphalium canescens DC., MG 416	Gordolobo	Bronchitis, expectorant	Flowers	Boiled, mixed with *Eucalyptus* leaves and honey bee
Gutierrezia sarothrae (Pursh) Britton & Rusby, MG 479	Escoba de cambray or escoba de rosita	Household goods	Dried branches	To make brooms
Matricaria recutita L., MG 417		Menstrual cramps	Leaves and branches	Boiled, drink as tea
		Intestinal colic	Leaves and branches	Boiled, drink as tea
		Constipation (children)	Leaves and branches	Boiled, drink as tea
		Conjuntivitis, cataracts	Leaves	Boiled, put eye drops
		Intestinal parasites	Leaves and branches	Boiled, mixed with *Artemisia ludoviciana, Prunus armeniaca, Mentha piperita* and *Chenopodium ambrosioides* leaves, drink as tea
		Burp	Leaves and branches	Boiled, drink as tea
		Respiratory diseases	Leaves and branches	Boiled, drink as tea
		Ceremonial	Complete plants	Bouquets
		Stomach pain	Leaves	Boiled, infusion

		Bronchitis, expectorant	Flowers	Boiled, drink as tea
		Ornamental	Complete plant	Planted in gardens
		Condiment and saborizant	Leaves and flowers	Boiled, drink as tea
Milleria quinqueflora L., MG 343	Cocolmeca	Lose weight	Dried root	Boiled, drink the solution
Tanacetum parthenium (L.) Sch.Bip. , MG 480	Altamisa	Stomach pain	Leaves	Boiled, infusion
Taraxacum officinale (L.) Weber ex F. H.Wigg., MG 494	Diente de león	Food	Leaves	Raw or cooked
		Anemia	Leaves	Raw or cooked, also, as infusion
		Diabetes	Leaves	Cooked
		Skin diseases	Complete plant	Ground, mixed with lemon, eat
		Vesicle	Leaves	Boiled, drink the infusion
Tagetes lucida (Sweet) Voss, MG 370	Yerbaníz	Intestinal colic	Complete plant	Boiled, drink infusion
		Colic cramps	Complete plant	Boiled, drink infusion
		Constipation	Complete plant	Boiled, drink infusion
		Bracing	Complete plant	Boiled, drink infusion
		Insect bites	Complete plant	Boiled, use as a poultice
		Condiment and saborizant	Leaves and flowers	Boiled
		Chest pain	Leaves	Infusion
		Cough	Leaves and flowers	Infusion
		Beverage	Leaves and flowers	Boiled, drink as tea
		Stomach pain	Leaves	Boiled, infusion
Tanacetum vulgare L., MG 359	Tanaceto	Ornamental	Complete plant	Planted in gardens
		Migraine	Leaves	Boiled, drink as tea
Tanacetum parthenium	Geranio de olor	Chest pain	Leaves	Boiled, drink as

(L.) Sch.Bip.,, MG 342				tea
Trixis californica Kellogg var. *californica*, MG 369	Árnica	Stomach ulcers	Flowers	Boiled, as infusion
		Against cancer	Flowers	Boiled, as infusión
		Inflammation	Flowers	Boiled, as infusion
		Acne	Leaves and flowers	Boiled, use as poultice (alcohol or water)
		Arthritis	Leaves	Boiled, as poultice
		Veterinary	Leaves and flowers	Ground and boiled, as infusión
		Ornamental	Complete plants	Planted in the gardens, beautiful flowers
		High pressure	Leaves and flowers	Boiled, drink as tea
		Intestinal colic	Complete plant	Boiled, drink as infusion
		Colic cramps	Complete plant	Boiled, drink as infusion
		Constipation	Complete plant	Boiled, drink as infusion
		High fever	Leaves	Boiled, drink as infusion
		Headache	Flowers	Boiled, drink as infusion
		Respiratory diseases	Leaves and branches	Boiled, drink as tea
Zinnia elegans L., MG 368	Cartulinas	Ornamental	Complete plants	Planted in the gardens, beautiful flowers
BALSAMINACEAE				
Impatiens balsamina L., MG 324	Belén	Ornamental	Complete plant	Planted in the gardens
Impatiens walleriana Hook.f., MG 367	Teresita	Ornamental	Complete plant	Planted in gardens
BEGONIACEAE				
Begonia uniflora S.	Begonia	Ornamental	Complete	Planted in gardens

Watson, MG 366			plant	
BIGNONIACEAE				
Chilopsis linearis (Cav.) Sweet, MG 495	Mimbre	Ornamental	Complete plant	Planted in gardens
Tecoma stans (L.) Juss. ex Kunth, MG 323	Tronadora, San Pedro	High pressure	Leaves and flowers	Boiled, drink as tea
		Diabetes	Leaves and flowers	Toasted, eat
		Ornamental	Complete plant	Planted in gardens
BORGINACEAE				
Borago officinalis L., MG 360	Borraja	High fever	Leaves	Infusion
		Measles	Leaves	Infusion; taking a bath with the solution
		Variecella	Leaves and branches	Infusion and poultice
		Remove phlegm	Leaves	Tea
Cordia boissieri A.DC., MG 365	Anacahuita	Respiratory diseases	Leaves and fruits	Mixed and boiled
		Tosferina	Flowers and fruits	Mixed and boiled, take in the morning and evenings
		Forage	Fruits	Raw
		Timber and fuel	Stems and braches	Dried
Ehretia anacua (Terán & Berland.) I. M. Johnst., MG 322	Anacua	Bunions and dewclaws	Leaves	Boiled, as poultice
		Food	Fruits	Raw or cook
		Timber, living fences, household good	Wood	Dried, planted.
		Forage	Fruits	Raw
Tiquilia canescens (A. DC.) A. T. Richardson, MG 496	Ventosidad	Colic	Leaves	Boiled, drink as tea
		Forage	Complete plant	Raw

BRASSICACEAE				
Lepidium virginicum L., MG 355	Hierba del pájaro	Cicatrizant	Leaves	Ground, spread in the affected area
Raphanus sativus L., MG 361	Rábano	Gallblader	Root	Raw or boiled, mixed with *Taraxacum officinale* leaves
Rorippa nasturtium-aquaticum (L.) Hayek, MG 497	Berro	Food	Leaves	Raw or cooked
		Anemia	Leaves	Raw or infusion
		Hepatitis	Leaves	Chewed or infusion
Sisymbrium irio L., MG 354	Mostaza	Laxative	Leaves	Chewed
		Headache	Leaves	Spread fat on leaves and stick them in occipital
BROMELIACEAE				
Ananas comosus (L.) Merr., MG 364	Piña	Food	Infrutescence	Raw
Hechtia scariosa L.B.Sm., MG 498	Guapilla	Fibers	Leaves	Scraped, fibers to make cords
		Lice control	Leaves	Toasted, ground, the ash resultant is smeared all over the head
Tillandsia bartramii Elliott, MG 331	Muérdago, paistle	Cictarizant	Leaves	Dried, ground, spread over the affected area
		Ornamental	Complete plant	In Christmas tree
BUDDLEJACEAE				
Buddleja cordata subsp. *tomentella* (Standl.) E. M. Norman, MG 330	Tepozán	Diabetes	Branches	Groun, boiled, driank as tea
		Remove ingrow toenails	Leaves	Boiled, as poultice over the affected area
		Smelly feet	Leaves	Boiled, mixed with *Trixis californica* leaves, wash feet with the solution

		Headache	Flowers	Ground, the pulp applyied directly on the occipital
CACTACEAE				
Ariocarpus scaphirostris Boed., MG 362	Cresta de gallos	Rheumatism	Complete plant	In alcohol, as poultice
Aztekium ritteri (Boed.) Boed., MG 354	Falso peyote	Arthritis	Complete plant	In alcohol, wait for nine months before use, then mix with garlic cloves, *Ruta chalepensis,Agave echuguilla* root, *Rosmarinus officinale* leaves, a quarter of avocado stone, applied as poultice in the affected area
Echinocactus platyacanthus Link & Otto, MG 363	Biznaga burra	Food	Fruits	"Cabuches", cooked, boiled with sugar to make candies
		Living fences	Stems	Planted in the gardens
		Ornamental	Complete plants	Planted in the gardens
		Forage	Stems	For goats, raw without spines
Echinocereus enneacanthus Engelm., MG 418	Alicoches	Sore tonsils	Fruits and stems	Toasted and placed as a poultice on the affected part
		Veterinary	Stems	The spines are removed, toasted and put on the legs broken as a substitute for plaster, bandage the leg
Echinocereus poselgeri Lem., MG 329	Sacasil	Burns	Root	Ground, as poultice in the

				affected area
		Cicatrizant	Root	Ground, as poultice in the affected area
		Veterinary	Root	Ground, as poultice, put on the legs broken as a substitute for plaster, bandage the leg
		Skin wounds	Root	Ground, mixed with soap, as poultice in the affected area
Ferocactus pilosus (Galeotti ex Salm-Dyck) Werderm. , MG 363	Biznaga roja	Food	Stems	Cooked
		To quench thirst	Fruits	Raw
		Ornamental	Complete plant	Planted in the gardens
Marginatocereus marginatus (DC.) Backeb., MG 419	Órgano	Food	Fruits	Raw or cooked
		Living fences	Complete plant	Planted in gardens
		Ornamental	Complete plant	Planted in gardens
Opuntia ficus-indica (L.) Mill., MG 328	Nopal de castilla	Food	Cladodes	Raw or cooked
		Diabetes	Cladodes	Liquefied, raw
		Cholesterol	Cladodes	Liquefied, raw
		Living fences	Complete plant	Planted in gardens
		Forage	Complete plant	Cut into pieces
		Ornamental	Complete plant	Planted in gardens
		Laxative	Cladodes	Liquefied, raw
		Lose weight	Cladodes	Liquefied, raw
Opuntia imbricata (Haw.) DC., MG 353	Coyonoxtle	Veterinary	Stems pulp	Raw or toasted, splinting broken limbs in humans

				or animals
		Inflamed tonsils	Fruit	Raw, ground, apply the pulp over the affected area
Opuntia leptocaulis DC.	Tasajillo	Food	Fruits	Raw or cooked
Opuntia lindheimeri Engelm., MG 258	Nopal serrano	Veterinary (broken limbs)	Cladodes pulp	Applied as poultice, splint and bandage the broken leg
		Retention	Root	Boiled, drink the solution
		Forage	Cladodes, flowers and fruits	Raw, cut into pieces
	Opuntia sp.	Forage	Complete plant	Raw, cut into pieces
CANNACEAE				
Canna indica L., MG 364	Coyol	Sunny	Root	Ground, spread over the entire head (people/animals)
		Ornamental	Complete plant	Planted in gardens
CARICACEAE				
Carica papaya L., MG 499	Papaya	Food	Fruit	Raw
CAPRIFOLIACEAE				
Lonicera japonica Thunb., MG 259	Madreselva	Ornamental	Complete plants	Planted in gardens, beautiful flowers
Sambucus candensis var. *mexicana* C. Presl., MG 327	Sauco	Flu	Branches	Boiled, drink as tea
		High fever	Branches and leaves	Boiled, drink as tea and also, take a bath with solution
		Cough	Flowers	Boiled, drink as tea
		Toothache	Leaves	Boiled in salt, gargle
CARYOPHYLLACEAE				
Dianthus deltoides L.,	Clavelinas	Ornamental	Complete	Planted in gardens

MG 420			plant	
CHENOPODIACEAE				
Beta vulgaris L., MG 341	Acelgas	Food	Leaves	Cook or raw
		Medicinal	Leaves	Infusion (anemia); cook (diabetes and blood pressure)
Chenopodium ambrosioides L., MG 260	Epazote	Intestinal parasites	Leaves and inflorescences	Boiled, mixed with leaves of *Matricaria recutita, Artemisia ludoviciana, Prunus persica* and *Mentha piperita*, drink the solution
		Condiment	Leaves	Mixed with frijoles (beans)
		Ceremonial	Complete plant	Dried, placed at the entrance of the doors of the houses
Salsola tragus L., MG 365	Capitana	Wounds	Leaves and stems	Toasted, wash the wound with the solution
COMMELINACEAE				
Commelina coelestis Willd., MG 352	Hierba del pollo	Dermic hemorrhages	Leaves	Ground, applied directly on the affected area}
Commelina dianthifolia Delile, MG 326	Hierba de la golondrina	Kidney diseases	Complete plant	Boiled, mixed with *Euphorbia glyptosperma* (leaves), *Zea mays* (styles), *Equisetum laevigatum* (stems), and *Mimosa malacophylla* (leaves), drink as tea
		Vomit	Branches	Boiled, drink the

				solution
		Cicatrize	Leaves	Crushed and mixed with cobweb, apply on the affected part
Tradescantia pringlei S. Watson, MG 340	Hierba de la gallina	High temperature	Leaves	Boiled, drink the solution
CONVOLVULACEAE				
Ipomoea batatas (L.) Lam., MG 399	Camote	Food	Root	Cooked
CANNABACEAE				
Cannabis indica Lam., (observed only)	Marihuana	Bruises	Leaves and branches	In alcohol, as poultice
		Joint pain	Leaves and branches	In alcohol, as poultice
		Migraine	Leaves and branches	In alcohol, as poultice
		Reumatism	Leaves and branches	In alcohol, as poultice
CRASSULACEAE				
Echeveria strictiflora A.Gray, MG 325	Siempreviva	Conjunctivitis	Leaves	Sap as a substitute for eye drops
		Ornamental	Complete plant	Planted in pots
Sedum diffusum S. Watson, MG 260	Chisme	Heart palpitations	Leaves	Raw, ground, chew
CUCURBITACEAE				
Citrullus lanatus (Thunb.) Matsum. & Nakai, MG 350	Sandía	Food	Fruit	Raw
Cucumis melo L.	Melón	Food	Fruits	Raw
Cucurbita ficifolia Bouché, MG 398	Chilacayote	Food	Fruit	Cooked or raw
		Wounds	Fruit	Toasted, apply the pulp directly in the affected part
Cucurbita foetidissima Kunth, MG 261	Calabacilla loca	Wash hair	Fruit and leaves	As a soap substitute, ground and in water
		Wash dishes	Fruit and leaves	As a soap substitute, ground

				and in water
		Vaginal cleansing	Fruit (mature)	Raw, put over the vagina after giving birth
Cucurbita moschata Duchesne, MG 366	Calabaza	Food	Flower; fruits; seeds	Cooked; cooked; raw (dried)
		Diabetes	Leaves and fruits	Cooked, mixed with nopales (*Opuntia* spp.)
Lagenaria siceraria (Molina) Standl., MG 324	Guaje or Huaje	Extract sap	Dried fruits	Cut at both ends, suction *Agave* sap
		Store water	Dried fruits	As a substitute canteen
CUPRESSACEAE				
Juniperus deppeana var. *deppeana* Steud., MG 397	Táscate	Timber and construction	Logs	Dried
		Ornamental	Complete plant	Planted in the gardens
Taxodium mucronatum Ten., MG 262	Sabino	Construction	Wood	Dried, logs
EQUISETACEAE				
Equisetum laevigatum A. Braun, MG 323	Cola de caballo	Cancer	Leaves	Boiled, drink the solution
		Prostate	Stems	Mixed with corn flower styles, boiled, drink the solution
		Kidney stones	Stems	Mixed with dried stem pieces of *Eysenhardtia texana*, boil and drink the solution
ERICACEAE				
Arbutus xalapensis Kunth, MG 262	Madroño	Food	Fruits	Raw
		Timber	Wood	Dried, in pieces
EUPHORBIACEAE				
Acalypha hederacea Torr., MG 367	Hierba del cáncer	Anticancer	Branches	Boiled, drink as tea
		Gastritis	Leaves and branches	Boiled, drink as tea

		Cough		Boiled, drink as tea
		Nosebleed	Branches	Boiled, inhale vapors arising boiled leaves
		Wounds		Boiled, as poultice
Bernardia myricifolia (Scheele) S. Watson, MG 368	Oreja de ratón	Diabetes	Leaves	Boiled, mixed with *Zea mays* styles and *Equisetum laevigatum* stems, drink as tea
Cnidoscolus chayamansa McVaugh, MG 421	Chaya	Diabetes	Leaves	Boiled, drink the solution
		Food	Leaves	Raw or boiled, to make salads
Croton suaveolens Torr., MG 396	Salvia	Amenorrhea	Stems and leaves	Boiled, drink as tea
		Anemia	Stems and leaves	Boiled, drink as tea
		Stomach disorders	Stems and leaves	Boiled, drink as tea
		Purify the blood	Stems and leaves	Boiled, drink as tea
		Bad breath	Stems and leaves	Boiled, drink as tea
Euphorbia antisyphilitica Zucc., MG 322	Candelilla	Veterinary (shed remnants of placenta)	Sap	Stems boiled (give drink to the animal a liter of the solution)
		Toothache	Stems	Boiled, swished around the mouth
		Protect grafts	Wax	Melted wax is spread on the graft union
Euphorbia glyptosperma Engelm., MG 321	Hierba de la golondrina	Kidney stones	Leaves	Boiled, mixed with *Commelian dianthifolia* (leaves), *Zea mays* (styles), *Equisetum laevigatum*

				(stems), and *Mimosa malacophylla* (leaves), drink as tea
		Hair loss	Root	Crushed, applied directly to the head
		Gingivitis	Root	Boiled, applied directly over the gums, and drink the solution as tea
Euphorbia pulcherrima Willd. ex Klotzsch, MG 320	Noche buena	Ornamental	Complete plant	Planted in gardens
Jatropha dioica Sessé, MG422	Sangre de drago	Urinary tract clean	Dried branches	Boiled, drink the solution
		Prostate	Dried branches	Boiled, drink the solution
		Avoid falling of teeth	Root	Chewed
		Cuts, grains, petty and warts	Sap	Spread the sap in the affected area
Tragia nepetifolia Cav., MG 263	Ortiguilla	Neutralize toxins (Ivy)	Leaves	Macerated, boiled, take a bath with the solution
		Good blood circulation	Leaves	Boiled, drink as tea
		Ceremonial	Complete plant	To purify the soul and remove the frighten
FABACEAE				
Acacia amentacea DC., MG 395	Chaparro prieto	Amoebiasis	Branches and leaves	Boiled, drink the infusion
		Gum diseases	Bark and branches	Swished around the mouth
		Shiny hair	Leaves and bark	Boiled, use as shampoo
		Household goods	Wood	Dried
		Fuel	Complete plant	Dried
		Ornamental	Complete plant	Planted in thegardens

Acacia berlandieri Benth., MG 423	Guajillo, sacuta	Forage	Leaves, buds and young branches	Raw
		Intestinal parasites	Branches	Pieces, dried, boiled, drink the solution
Acacia farnesiana (L.) Willd., MG 272	Huizache	Inflammation	Bark (ground) and leaves	Boiled, mixed with *Agave bracteosa* root (ground and dried),drink as tea
		External shocks	Bark (ground) and leaves	Boiled, mixed with *Agave bracteosa* root (ground and dried),drink as tea
		Forage	Leaves and fruits	Raw
		Construction	Wood	Dry, in pieces
Arachis hypogaea L., MG 501	Cacahuate	Food	Fruit	Raw, toasted
Astragalus sanguineus Rydb., MG 508	Garbancillo	Ornamental	Complete plant	Planted in gardens
Caesalpinia mexicana A. Gray, MG 265	Hierba del potro	Tootache	Leaves	Ground and boiled, swished around the mouth
Bauhinia purpurea L., MG 424	Pata de vaca	Ornamental	Complete plant	Planted in gardens
Havardia pallens (Benth.) Britton & Rose, MG 264, MG 319	Tenaza	Timber	Wood	Logs
		Construction	Wood	Dry pieces
		Forage	Leaves	Raw
		Household goods	Wood	Dry (hoe handles)
		Fences	Wood	Dry branches
		Charcoal	Wood	To cook food
Medicago sativa L., MG 394	Alfalfa	Forage	All plant	Dry, raw
Eysenhardtia texana Scheele, MG 512	Vara dulce, palo azul, vara	Purify the blood	Wood (small pieces)	Dry pieces in water for three

	azul			days, not boil, use the solution as daily, drink it for four consecutive days
		Diabetes	Wood (small pieces)	Dry pieces in water for three days, not boil, use the solution as daily, drink it for four consecutive days
		Kidney diseases	Wood (small pieces)	Dry pieces in water for three days, not boil, use the solution as daily, drink it for four consecutive days
		Varicella	Wood (small pieces)	Dry pieces in water for three days, not boil, use the solution as daily, drink it for four consecutive days
		Gallblader	Wood (small pieces)	Dry pieces in water for three days, not boil, use the solution as daily, drink it for four consecutive days
Mimosa malacophylla A. Gray, MG 318	Charrasquilla	Kidney diseases	Root	Boiled, drink the solution
		Backpains	Root	Boiled, drink the solution
		Mumps	Fruits	Toasted, apply directly to the affected area
Mimosa pudica L., MG 271	Sensitiva, vergonzosa	Ornamental	Complete plant	Planted in Gardens
Phaseolus vulgaris L., MG 425	Frijol	Food	Fruit	Cooked

		Veterinary	Immature fruits	Mixed with leaves of *Mentha piperita*, to shed the remnant of the placenta
Pithecellobium dulce (Roxb.) Benth., MG 317	Guamuchil	Flu	Leaves	Boiled, drink as tea
Prosopis glandulosa var. *torreyana* (L. D.Benson) M. C. Johnst., MG 267	Mezquite	Cataracts	Leaves	Ground, fermented in water, put eye drops
		Timber, charcoal	Wood	Dried
		Living fences	Complete plant	Planted in the yard
		Forage	Fruits	Dried, to feed pigs
		Food	Fruits	Raw or cooked
		Make chees	Fruits	Boiled with milk
		Veterinary	Resin	Boiled, mixed with ground bark, spread and leg splints
		Ceremonial	Wood	Burned, the frgihtned person should smell the smoke, making a cross with the hands
FAGACEAE				
Quercus polymorpha Schltdl. & Cham., MG 393	Encino	Forage	Fruits	Raw, for pigs mainly
		Food	Fruits	Ground and mixed with cornmeal, to make tortillas
		Gingivitis	Bark	Ground, swish
		Toothache	Bark	Ground, swish
		Timber	Wood	In pieces
Quercus emoryi Torr., MG 392	Encino	Food	Fruits	Ground and mixed with

				cornmeal, to make tortillas
		Pyorrhea		Ground, swish
		Gingivitis	Bark	Ground, swish
		Toothache	Bark	Ground, swish
		Timber	Wood	In pieces
FOUQUIERIACEAE				
Fouquieria splendens Engelm., MG 426	Ocotillo, albarda	Tooth ache	Leaves	Raw, chewed
		Colic	Leaves	Raw, chewed
		Stomach ache	Leaves	Boiled, drink as tea
		Living fences	Complete plant	Planted in gardens
		Construction	Dried branches	To make animal pens
		Fuel	Complete plant	Dried parts
		Forage	Leaves and flowers	Raw
GERANIACEAE				
Pelargonium x hortorum L.H. Bailey, MG 268	Geranio	Ornamental	Complete plant	Planted in gardens, multicolor flowers
		Burp	Leaves	Boiled, drink as tea
		Stomach ache	Leaves	Boiled, mixed with leaves of *Litsea glauscecens*
		Constipation (suppository)	Petiole	Lubricated with fat, anally inserted in children
		Inflammation	Leaves	Boiled, solution applied as poultice in the affected area
HYDRANGEACEAE				
Hydrangea macrophylla (Thunb.) Ser., MG 427	Hortensia	Ornamental	Complete plant	Planted in gardens
IRIDACEAE				
Gladiolus grandiflorus Andrews, MG 349	Gladiola	Ornamental	Complete plant	Planted in gardens,

				multicolor flowers
JUGLANDACEAE				
Carya illinoinensis (Wangenh.) K.Koch, MG 282	Nogal	Gingivitis	Leaves	Boiled, drink as tea
		Diuretic	Leaves	Boiled, drink as tea
		Lubricate joints	Leaves	Boiled, drink as tea
		Healthy heart	Leaves	Boiled, drink as tea
		Harden gums (prevent tooth fall)	Leaves and bark	Boiled, leaves and bark, drink as tea, or also, chew the bark
		Anemia	Leaves and bark	Boiled, drink as tea
		Constipation	Leaves	Dry, ground, mixed with glycerine into a paste, rub in the stomach
		Food	Fruits	Raw
		Ceremonial	Leaves	Smoked, to purify the soul
		Construction	Wood	Logs
		Hair dye	Fruit peel	Boiled, it turns dark-brown or almost black, dyeing hair with the solution
Juglans mollis Engelm., MG 269	Nogal criollo	Food	Fruit	Raw (pecan halves)
		Anemia	Leaves and bark	Boiled, drink as tea
		Healthy blood	Leaves and bark	Boiled, drink as tea
		Shampoo	Bark	Ground, mixed with *Agave 25echeguilla* root , *Jatropha dioica* root, *Rosmarinus officinale* leaves,

				Aloe vera pulp, *Equisetum laevigatum* stems (ground), *Melissa officinalis* leaves (ground), and *Carya illinoiensis* bark, boil them together, strain the contents and use it as shampoo
		Ceremonial	Leaves	Smoked, to purify the soul
		Timber	Wood	Dry, in pieces
		Living fences	Complete plant	Planted in gardens
		Construction	Wood	Logs
		Fuel	Wood	Dry wood
		Ornamental	Complete plant	Planted in gardens
LAMIACEAE				
Aloysia triphylla (L'Hér.) Britton, MG 348	Cedrón de Castilla	General pains	Leaves	Drink the solution
		Hepatitis	Leaves	Boiled, drink as tea
Hedeoma drummondii Benth., MG 270	Poleo	Abortive	Leaves	Boiled, drink as tea
		Intesitnal colic	Leaves	Boiled, drink as tea
		Expectorant	Leaves	Boiled, drink as tea
		Flatulences	Leaves	Boiled, drink as tea
		Insomnia	Leaves	Boiled, drink as tea
		Burp	Leaves	Boiled, drink as tea
		Relaxing	Leaves	Boiled, drink as tea
		Ceremonial	Complete plant	Dried, bouquets, along with *Litsea glauscecens* and *Matricaria recutita* dried

				plants, and *Brahea duclcis* (leaves)
Majorana hortensis Moench, MG 347	Mejorana	Laxative	Branches and leaves	Drink as tea
		Intestinal colic	Branches and leaves	Drink as tea
		Condiment	Leaves	Raw or cooked, mixed with *Ocimum basilicum* leaves
Marrubium vulgare L., MG 316	Marrubio, manrrubio or marruño	Amebiasis	Branches and leaves	Boiled, drink as tea
		Lose weight	Branches and leaves	Boiled, drink as tea
		High pressure	Branches and leaves	Boiled, drink as tea
		Gallbladder pain	Branches and leaves	Boiled, drink as tea
		Stomach ache	Branches and leaves	Boiled, drink as tea
		Diabetes	Branches and leaves	Boiled, drink as tea
		Nervousness	Branches and leaves	Boiled, drink as tea
		Flu	Branches and leaves	Boiled, drink as tea
		Constipation	Branches and leaves	Boiled, drink as tea
		Alopecia	Branches and leaves	Boiled, spread over the head
		Ceremonial	Complete plant	Bouquets
Melissa officinalis L., MG 346	Citronela, toronjil	Aroma	Leaves	Add boiled leaves to shampoo
		Heartache	Branches (small pieces)	Boiled, drink as tea
		Stomach ache	Leaves	Boiled, mixed with leaves of *Ocimum basilicum*, drink as tea
		Lice control	Leaves	Mixed with bone

				mashed avocado, *Ruta chalepensis* and *Chenopodium ambrosioides*, leaves, boil and strain the mixture, use as shampoo
Mentha x piperita L., MG 315	Hierbabuena	Intestinal colic	Leaves	Boiled, drink as tea
		Constipation	Leaves	Boiled, drink as tea
		Intestinal parsites	Leaves	Boiled, drink as tea
		Bad breath	Leaves	Chew the leaves
		Beverage	Leaves	Boiled, drink as tea
		Ceremonial	Complete plant	Shaking the plant throughout the body
		Ornamental	Complete plant	Planted in gardens
		Relaxing	Leaves	Boiled, drink as tea
		High pressure	Leaves	Boiled, drink as tea
		Headache	Leaves	Boiled, drink as tea
		Laxative	Leaves	Boiled, drink as tea
		Flatulences	Leaves	Boiled, drink as tea
		Cough	Leaves	Boiled, mixed with honey bee and lemon leaves, drink as tea
		Menstrual cramps	Leaves	Boiled, drink as tea
Monarda citriodora var. *austromontana* (Epling) B. L.Turner, MG 390	Betónica	Urinary system	Leaves	Infusion
		Backache	Leaves	Infusion
		Menstrual cramps	Leaves, branches	Infusion
		Eye infection	Leaves	Put eye drops

		Beverage	Leaves	Drink, delicious taste
		Flatulences	Leaves	Boiled, drink as tea
		Cough	Leaves	Boiled, mixed with honey bee and lemon leaves, drink as tea
		Ceremonial	Complete plant	Shaking the plant throughout the body
		Ornamental	Complete plant	Planted in gardens
		Relaxing	Leaves	Boiled, drink as tea
		Intestinal colic	Leaves	Boiled, drink as tea
		Constipation	Leaves	Boiled, drink as tea
		Intestinal parsites	Leaves	Boiled, drink as tea
		Bad breath	Leaves	Chew the leaves
		Respiratory diseases	Leaves	Infusion
Ocimum basilicum L., MG 345	Albahacar	Intestinal colic	Leaves	Infusion
		Respiratory diseases	Leaves	Infusion
		Stomach pain	Leaves	Infusion
		Relaxing	Leaves	Infusion
		Cough	Leaves	Infusion
		Tachycardia	Leaves	Infusion
		Colitis	Leaves	Infusion
		Chest pain (heart)	Leaves	Mixed with canela
		Remove bad luck	Complete plant (dried)	Above of the main entrance door of the house
		Inflammation	Leaves	Boiled, drink as tea
Rosmarinus officinalis L., MG 314	Romero	Cicatrizant	Branches and leaves	Ground, applying on the affected part

		Migraine	Leaves	Boiled, drink as tea
		Inflammation	Leaves	Boiled, drink as tea
		Skin infections (grains)	Leaves and branches	Boiled, as poultice, applying directly on the affected area
		Cough	Leaves	Boiled, drink as tea
		Beverage	Leaves	Boiled, drink as tea
		Insomnia	Leaves	Boiled, drink as tea, and also, put dried leaves below pillow when sleeping
		Colitis	Leaves	Boiled, drink as tea
		Dizziness	Leaves	Boiled, drink as tea
		High pressure	Leaves	Boiled, drink as tea
		Memory	Leaves	Boiled, drink as tea
		Colic	Leaves	Boiled, drink as tea
		Headache	Branches	Ground, in water, gargle
		Firming skin	Branches and leaves	Boiled, wash the body with the solution
		Shocks	Branches and leaves	Boiled, as a poultice, applying in the affected area
		Smelly feet	Branches and leaves	Ground, put into the shoes
		Shiny hair	Leaves and branches	Ground, in water, wash the hair with the solution
		Ceremonial	Complete plant	Purify the soul

		Frighten	Complete plant	Dried, pass the plant on the body
		Ornamental	Complete plant	Planted in gardens
Salvia microphylla Kunth, MG 312	Mirto	Antibiotic (children	Complete plant	Boiled, take a bath with solution
		Constipation (children)	Leaves	Boiled, drink as tea
		Ceremonial (frigth)	Complete plant	Pass the plant on the body
		Ornamental	Complete plant	Planted on gadrens
Thymus vulgaris L., MG 389	Tomillo	Condiment	Leaves	Ground, raw
		Expectorant	Branches (small pieces)	Boiled, drink as tea
LAURACEAE				
Cinnamomum verum J. Presl., MG 388	Canela	Intestinal colic, colic cramps	Bark	Infusion
		Condiment, flavoring	Bark	Boiled
Litsea glaucescens Kunth, MG 428	Laurel	Chest pain	Leaves	Boiled, mixed with canela bark and orange peel, drink the solution
		Bone pain	Leaves	Boiled, drink as tea
		Menstrual cramps	Leaves	Boiled, drink as tea
		Stomach ache	Leaves	Boiled, drink as tea
		Flatulency	Leaves	Boiled, drink as tea
		Laxative	Leaves	Boiled, drink as tea
		Relaxing	Leaves	Boiled, drink as tea
		Reumatism	Leaves	Boiled, drink as tea
		Backache	Leaves	Boiled, drink as tea
		Condiment	leaves	Boiled

		Ceremonial	Complete plant	Bouquets, dried plants of laurel, *Hedeoma drummondii*, *Matricaria recutita* and *Brahea dulcis*
		Expectorant	Leaves	Infusion
		Cough	Leaves	Infusion
		High pressure	Leaves	Boiled, drink as tea
		Relaxing	Leaves	Infusion
Persea americana var. *drymifolia* (Cham. & Schltdl.) S. F. Blake, MG 344	Aguacate	Respiratory diseases	Leaves	Infusion
		Expectorant	Leaves	Infusion
		Cough	Leaves	Infusion
		Intestinal parasites	Leaves	Infusion
		Amenorrhea	Leaves	Infusion
		Menstrual cramps	Leaves	Infusion
		Constipation	Leaves	Infusion
		Hernia	Leaves	Infusion
		Relaxing	Leaves	Infusion
		Bracing	Leaves	Infusion mixed with milk
		Surfeit	Seed	Ground
		Food	Fruit	Raw
Sassafras albidum (Nutt.) Nees, MG 311	Sasafrás	Anemia	Leaves	Boiled, drink as tea
		Kidney diseases	Leaves and bark	Boiled, drink as tea
		Hardening of the skeletal system	Bark and leaves	Boiled, drink as tea
		Gallblader	Leaves	Boiled, drink as tea
		Purify the blod	Bark	Boiled, drink as tea
LILIACEAE				
Allium cepa L., MG 343		Food	Stems	Raw or cooked

		Acne	Stems	Boiled, the bulb layers, put them in the affected area
		Indigestion (children)	Stems	Spread in the gum to avoid hindrance
		Bronchitis	Stems	Toasted or boiled, drink the solution
		Give up smoking	Stems	Chew two bulbs mixed with lemon and honey bee, strain and drink
Allium sativum L., MG 310	Ajo	Cough	Leaves	Infusion or tea
		Varices	Leaves	Tea
		Insect bites	Leaves	Boiled, poultice
		Gastritis	Two cloves	Mixed with onion, lemmon coriander, take half tea spoon every morning
		Arthritis	Cloves	Chewing 1 raw clove
		Constipation	3 cloves	Boiled, drink the water
		Condiment	Cloves	Cook
Lilium longiflorum Thunb., MG 273	Lirio	Ornamental	Complete plant	Planted in gardens
LYTHRACEAE				
Heimia salicifolia (Kunth) Link, MG 274	Jarilla de río	Household goods	Dried stems and branches	To make brooms
MALVACEAE				
Althaea rosea (L.) Cav., MG 429	Malvarosa	Ornamental	Complete plant	Planted in gardens, beautiful flowers
Hibiscus campanulifolius Ulbr., MG 329	Tulipán	Ornamental	Complete plant	Planted in gardens
Malva parviflora L., MG 342	Malva de marrano	Intestinal colic	Leaves	Boiled, drink as tea
		Menstrual cramps	Leaves	Boiled, drink as tea
		Constipation	Leaves	Boiled, drink as tea

		Expectorant	Leaves	Boiled, drink as tea
		Flu	Leaves	Boiled, drink as tea
		Laxative	Branches	Boiled, drink as tea
		Forage	Complete plant	Dry, raw
MELIACEAE				
Azadirachta indica A. Juss., MG 387	Neem	Diabetes	Leaves	Boiled, drink as tea
MORACEAE				
Ficus carica L., MG 331	Higuera	Food	Infrutescences	Raw or cooked
		Constipation	Leaves	Boiled, half leaf per cup
		Cough	Leaves	Boiled, drink as tea
		Intestinal parasites	Leaves	Boiled, drink as tea
		Promote milk production in women	Leaves	Boiled as tea
Morus celtidifolia Kunth, MG 332	Mora	Food	Fuits	Raw
		Hair health	Leaves	Ground, mixed with *Fraxinus 34uspidate* and *Glandularia bipinnatifida*, wash the hair with the solution
MUSACEAE				
Musa x paradisica L., MG 290	Plátano	Vaginal infection	Peel	Ground, mixed with two tablespoons of vinegar, applying directly on the affected part
		Food	Fruit	Raw
MYRTACEAE				
Eucalyptus globulus Labill., MG 386	Eucalito	Respiratory diseases or expectorant	Leaves	Boiled, drink as tea, or as poultice
Psidium guajava L., MG	Guayaba	Food	Fruit	Raw

521				
		Stomach ache	Leaves	Boiled, drink as tea
NEPHROLEPIDACEAE				
Nephrolepis exaltata (L.) Schott, MG 522	Helecho	Ornamental	Complete plant	Planted in gardens
NOLINACEAE				
Dasylirion texanum Scheele, MG 333	Sotol	Forage	Inflorescences	Raw
		Ornamental	Complete plant	Planted in gardens
NYCTAGINACEAE				
Bougainvillea spectabilis Willd., MG 451	Bugambilia	Ornamental	Complete plant	Planted in the gardens, beautiful colored bracts
OLEACEAE				
Fraxinus cuspidata Torr., MG 430	Fresno silvestre	Headache	Leaves	Boiled, mixed with leaves of *Salix bonplandiana*, drink the solution
		Ornamental	Complete plant	Planted in gardens
		Shampoo	Leaves	Mixed with leaves of *Morus celtidifolia*, wash the head with the solution
Fraxinus greggii A. Gray, MG 340	Barreta verde	Toothache	Leaves	Boiled, swish around
Jasminum floridum Bunge, MG 431	Jazmín	Ornamental	Complete plant	Planted in gardens
Ligustrum japonicum Thunb., MG 289	Trueno	Ornamental	Complete plant	Planted in gardens
Olea europaea L.	Olivo	Cholesterol	Leaves	Boiled, drink as tea
ONAGRACEAE				
Oenothera rosea L'Hér. ex Aiton, MG 341	Hierba del golpe	Hematoma	Leaves and branches	Boiled, as poultice
		Inflammation		Boiled, as poultice
		Stomach disorders	Branches and leaves	Boiled, drink as tea
PAPAVERACEAE				

Argemone mexicana L., MG 385	Cardo	Warts and petty	Sap	Apply the sap in the affected area
PICRAMNIACEAE				
Picramnia sp., MG 334	Palo mulato	Asthma	Leaves and bark	Boiled, drink as tea
		Expectorant	Leaves	Boiled, drink as tea
		Body aches	leaves	Boiled, drink as tea
		Skin infections	Leaves and sap	Boiled, as poultice
PINACEAE				
Pinus cembroides Zucc., MG 339	Pino piñonero	Veterinary	Resin	Melted, spread on broken limbs in animals, splinting and bandaging
		Grain	Resin	Applied directly over the grain
		Thorns within the skin	Resin	Melted, applied directly over the affected area to extract it
		Construction	Wood	Logs
		Fuel	Wood	Dried pieces
		Timber	Wood	Wood, in pieces
		Food	Seed (piñones)	Raw
		Protect grafts (avocado)	Resin	Melted resin is spread on the graft union
PLANTAGINACEAE				
Plantago major L., MG 432	Llantén or Quelite de llantén	Hemorrhoids	Leaves	Boiled, apply the solution in the affected área
		Antibiotic	Leaves	Boiled, drink the solution
		Ingrown toenails	Leaves	Boiled, as poultice
POACEAE				
Arundo donax L., MG 335	Carrizo	Construction	Stems	Dried
		Living fences	Complete plant	Planted in the gardens

		Household goods	Stem strips	To make brooms
		Ornamental	Complete plant	Planted in the gardens
Avena fatua L., MG 384	Avena	Forage	Complete plant	Raw
Cymbopogon citratus (DC.) Stapf., MG 338	Zacte limón	Respiratory diseases	Leaves and stems	Boiled, drink as tea
		Cancer	Leaves and stems	Boiled, drink as tea
		Kidney diseases	Leaves and stems	Boiled, drink as tea
		Food	Leaves and stems	Boiled, drink as tea
Hordeum vulgare L., MG 288	Cebada	Pertussis (tosferina)	Leaves and stems	Boiled, drink solution
		Forage	Leaves and stems	Raw
		Construction	Leaves and stems	Mixed with mud to make bricks
Saccharum officinarum L., MG 336	Caña	Food	Sap	Dried
		Candies	Sap	Melted with piloncillo and pecan halves
Sorghum halepense (L.) Pers., MG 531	Sorgo	Forage	Complete plant	Dry, raw
Triticum aestivum L., MG 337	Trigo	Food	Seeds	Ground, to make tortillas
		Forage	Complete plant	Dry, raw
Zea mays L., MG 433	Maíz	Kidney diseases	Female flowers	Styles, boiled, drink as tea
		Food	Fruits	Boiled, cooked, toasted
		Food	Bracts	Dried, used to make tamales
		Forage	Leaves, stems	Raw, ground
POLEMONIACEAE				
Loeselia mexicana (Lam.) Brand, MG 383	Hierba de la virgen	Estrogen productiom	Branches	Boiled, drink as tea

PORTULACACEAE				
Portulaca mundula I. M. Johnst., MG 275	Verdolaga, amores, amor de un rato	Ornamental	Complete plant	Planted in gardens
		Food	Complete plant (without roots)	Cooked
		Anemia	Leaves	Boiled or cooked
		Inflammation	Branches and leaves	Boiled, drink as tea
PUNICACEAE				
Punica granatum L., MG 434	Granada	Food	Fruit	Raw
		Intesitnal parasites	Seeds	Boiled, drink the solution
		Hemorrhage	Fruit peel	Boil, together with nutshell, drink the solution and also, apply as poultice
		Skin irritation and allergies (Ivy, *Tragia 38amose*)	Leaves	Boiled, take a bath with the solution
RANUNCULACEAE				
Delphinium madrense S. Watson, MG 458	Espuela de caballero	Ornamental	Complete plant	Planted in gardens
RHAMNACEAE				
Karwinskia humboldtiana (Schult.) Zucc., MG 287	Coyotillo, tullidora	Headache (migraine)	Leaves	Ground, as poultice on the head
ROSACEAE				
Crataegus greggiana var. *greggiana* Eggl., MG 278	Tejocote	Food	Fruit	Raw or cooked
		Cholestrero	Branches and leaves	Boiled, drink the solution
Cydonia oblonga Mill., MG 435	Membrillo	Food	Fruits	Raw or cooked (canned fruits)
		Alcoholic beverages	Fruits	Fermented
		Ornamental	Complete plant	Planted in gardens

Eriobotrya japonica (Thunb.) Lindl., MG 369	Níspero	High pressure	Leaves	Boiled, drink as tea
		Diabetes	Leaves	Boiled, drink as tea
		Food	Fruits	Raw
Fragaria vesca L., MG 382	Fresa	Food	Fruits	Raw
Lindleya mespiloides Schltdl., MG 381	Pinacate	Skin infections	Bark and leaves	Boiled, applied as poultice
		Cancer prevention	Leaves	Boiled, drink as tea
Malus domestica Borkh., MG 436	Manzana	Food	Fruits	Raw or cooked
		Alcoholic beverages	Fruits	Fermented
		Ornamental	Complete plant	Planted in gardens
		Ornamental	Complete plants	Planted in gardens
Prunus armeniaca L., MG 370	Chabacano	Food	Fruit	Raw or cooked
		Ornamental	Complete plant	Planted in gardens
Prunus domestica L., MG 371	Ciruelo	Food	Fruit	Raw or cooked
		Constipation	Fruit	Cooked
Prunus persica (L.) Batsch, MG 372	Durazno	Food	Fruit	Raw or cooked
		Grains and skin irritation	Branches	Boiled, apply as a poultice
		Intestinal parasites	Leaves	Boil them mixed with *Matircaria recutita*, *Artemisia ludoviciana*, *Mentha piperita* and *Chenopodium ambrosioides* leaves, drink the solution during nine consecutive days
		Worms in the skin of animals	Leaves	Mixed with lime, spread in the

				affected part
		Epidermal wounds	Leaves	Boiled, mixed with *Opuntia 40exicana40* toasted pulp and oil, spread over the affected area
Prunus serotina ssp. *capuli* (Cav. ex Spreng.) McVaugh, MG 380	Capulín	Anemia	Bark	Boiled, drink solution
		Food	Fruits	Raw
		Alcoholic beverages	Fruits	Fermented
				Infusion
				Infusion
Pyrus communis L., MG 279	Pera	Food	Fruits	Raw or cooked
		Ornamental	Complete plant	Planted in gardens
Rosa gallica L., MG 459	Rosal	Ornamental	Complete plant	Planted in gardens
Rosa montezumae Humb. & Bonpl. ex Redout & Thory, MG 379	Rosa de castilla	Intestinal colic	Leaves	Boiled, drink as tea
		Diabetes	Leaves	Boiled, drink as tea
		Gastritis	Leaves	Boiled, drink as tea
		Constipation (children)	Petals	Boiled, mixed with *Matricaria recutita* and *Artemisia ludoviciana* leaves, drink as tea
		Stomach ache	Leaves	Boiled, drink as tea
		Inflammation	Leaves	Boiled, drink as tea
		Food	Petals	Raw (sweet flavor)
Rubus aff. *humistratus* Steud., MG 280	Zarzamora	Food	Fruits	Raw

RUTACEAE				
Casimiroa greggii (S. Watson) F. Chiang, MG 410	Manguito, zapote blanco	Cholesterol	Leaves	Boiled, drink as tea
		Insomnia	Leaves	Boiled, drink as tea
Citrus paradisi Macfad., MG 460	Toronja	Food	Fruits	Raw
Citrus limon (L.) Osbeck, MG 378	Limón	Food	Fruits	Raw
		Sore tonsils	Leaves	Boiled, drink as tea
		Expectorant	Fruits	Juice mixed with canela bark
		Cataracts	Fruits	Add lemon drops in eyes
		Purify blood	Fruits	Lemon juice for nine days on an empty stomach
Citrus nobilis Lour., MG 461	Mandarina	Food	Fruits	Raw
Citrus sinensis (L.) Osbeck, MG 411	Naranja	Food	Fruits	Raw
		Insomnia	Leaves	Boiled, drink as tea
		Nervousness		Boiled, drink as tea
		Condiment		Orange juice, used to seasoning the meat
		Kidney diseases	Leaves	Boiled, drink as tea
Helietta parvifolia (A. Gray) Benth., MG 377	Barreta	Construction and timber	Stems and branches	Dried
		Fences	Stems and branches	Dried
		Fuel	Stems and branches	Dried
		Household goods	Stems and btranches	Dried
		Toothache	Leaves and bark; Leaves	Chew or boiled,drink as

				infusion
		Prostate	Leaves and bark	Boiled, drink as tea every four hours for a week
Ruta chalepensis L., MG 281	Ruda	Abortive	Leaves	Boiled, drink the solution
		Colic	Leaves	Boiled, drink the solution
		Rheumatism	Leaves	Boiled, as poultice, applying directly on the affected area
		Artritis	Branches and leaves	Into alcohol, as poultice, applying directly on the affected area
		Earache	Leaves	Boiled, put drops in the ear hole
		Breast firming	Leaves	Boiled, applying the solution directly on the breast
		Skin infections	Leaves	Boiled, as poultice, applying directly on the affected area
		Ceremonial	Complete plant	For good luck
		Frigthen	Complete plant	Pass the plant on the body
		Ornamental	Complete plant	Planted in gardens
Zanthoxylum fagara Marshall, MG 412	Colima	Numb gums	Dried branches	Boiled, drink the solution
		Constipation in children	Leaves and branches	Boiled, drink the solution
		Upset stomach	Branches	Boiled, drink the solution
		Rheumatism	Branches	Boiled, apply as poultice
SALICACEAE				
Populus deltoides Marshall, MG 437	Álamo	Ornamental	Plant	Planted in the gardens
		Veterinary	Leaves	Boiled with

				manzanilla, and hierbabuena; freshly calved animals, to shed the remnants of placenta
Salix humboldtiana Willd., MG 438	Sauce	Timber	Wood	Logs
		Ornamental	Complete plant	Planted in gardens
		Healthy hair	Leaves	Boiled, mixed with *Glandularia bipinnatifida* and *Fraxinus cuuspidata* leaves, wash the hair with the solution
		Ceremonial	Branches and leaves	Dried, pass the plant on the body
SAPINDACEAE				
Dodonaea viscosa (L.) Jacq., MG 376	Jarrón	Alergies	Leaves	Boiled, as poultice, applied in the affected part
Koelreuteria paniculata Laxm., MG 356	Alfombrilla	Ornamental	Complete plant	Planted in the gardens
SAPOTACEAE				
Sideroxylon lanuginosum Michx., MG 523	Coma	Food	Fruits	Chew as chewing gum
		Beverage	Fruits	Ground, mixed with water, as softdrink
SCROPHULARIACEAE				
Leucophyllum frutescens (Berland.) I. M. Johnst., MG 282	Cenizo	Kidney diseases	Leaves and branches	Boiled, drikn the solution
		Cough	Leaves	Infusion
		Hepatitis	Leaves	Wahes and infusion and, add aslo dried branches and leaves in the bed and under the pillow and sleep

				on them
		Jitters	Leaves	Rub the plant on the body
		Ceremonial	Dried plant	Rub the dried plant on the body
		Living fences	Complete plant	Planted in gardens
		Skin diseases	leaves	Boiled with salt, take three baths at day
		Ornamental	Complete plant	Planted in the gardens
		Forage	Leaves and flowers	Dried, raw
SELAGINELLACEAE				
Selaginella pilifera A. Braun, MG 357	Flor de peña	Cleaning of urinary tract	Leaves	Boiled, drink as tea
SIMAROUBACEAE				
Castella erecta subsp. Texana (Torr. & A. Gray) Cronquist, MG 439	Bisbirinda	Amoebiasis	Leaves and stems	Infusion, 4-5 leaves only, has a very bitter taste and it astringent
		Anticancer	Leaves	Infusion
SIMMONDSIACEAE				
Simmondsia chinenis Nutt., MG 358	Jojoba	Shampoo	Root, Sap	Ground, spread on the hair
SMILACEAE				
Smilax bona-nox L., MG 440	Zarzaparrilla	Blood circulation, purify the blood	Root	Ground, boiled, drink as tea
		Grains	Root	Ground, boiled, drink as tea
SOLANACAEAE				
Capsicum annuum var. *glabriusculum* (Dunal) Heiser & Pickersgill, MG 376	Chile piquín	Condiment	Fruit	Food flavoring, raw or cooked
Capsicum frutescens L., MG 524	Chile japonés	Condiment	Fruit	Food flavoring, raw or cooked
Datura stramonium L., MG 283	Toloache	Make a person fall in love with me	Leaves and seeds	Ground, spread in the food
Lycopersicon esculentum Mill., MG 525	Tomate cherry	Food	Fruit	Raw or cooked

Nicotiana glauca Graham, MG 284	Gigante	Headache, migriane	Leaves	Spread with fat and place in the parietal
Nicotiana tabacum L., MG 467	Tabaco	Smoke	Leaves	Dry, ground
Physalis philadelphica Lam., MG 359	Tomate de fresadilla, tomatillo	Food and condiment	Fruit	Raw or cooked
Solanum douglasii Dunal, MG 375	Hierba del ratón	Rat poison	Leaves	Ground, spread on floor
Solanum elaeagnifolium Cav., MG 466	Trompillo	Cataracts	Seeds	Ground, applying directly into eyes
Solanum tuberosum L., MG 360	Papa	Food	Roots	Cooked
		gastritis	Roots	Liquefied, raw
TROPAEOLACEAE				
Tropaeolum majus L., MG 285	Mastuerzo	Headache	Leaves	Ground, mixed with Vaporub and spread in the occipital
TURNERACEAE	Damiana, yeba del venado	Colic cramps	Leaves	Boiled, drink the solution
Turnera diffusa Willd., MG 374		Diabetes	Leaves and flowers	Boiled, drink the solution
		Stimulate fertilty in women	Leaves, stems and flowers	Boiled, drink the solution
		Intestinal infection	Leaves	Boiled, drink the solution
		Insomnia	Leaves	Boiled, drink the solution
		Backache	Leaves	Boiled, drink the solution
		Impotence	Dried flowers and leaves	Mixed with dried leaves of *Chrysactinia mexicana*, boiled, drink the solution
		High pressure	Leaves	Boiled, drink the solution
		Kidney diseases	Leaves	Boiled, drink as tea
		Matrix inflamated	Leaves	Boiled, drink as tea

		Headache	Root	Ground, boiled, drink the solution
		Skin diseases	Branches	Boiled, drink as tea
		Grains	Branches	Boiled, drink as tea
		High fever	Branches	Boiled, drink as tea
		Ornamental	Complete plant	Planted in gardens
TYPHACEAE				
Typha latifolia L., MG 465	Tule	Handcrafts	Leaves and stems	Fresh tissues, easier to work with the hands (if leaves and stems are dry, the handraft could break)
ULMACEAE				
Celtis laevigata Willd, MG 360	Palo blanco	Timber	Wood	Pieces
		Fences	Wood	Pieces
		Fuel	Wood	Dried
Celtis pallida Torr., MG 526	Granjeno	Food	Fruit	Raw or as syrup
		Forage	Leaves	Raw
		Timber	Wood	Dried pieces
USTILAGINACEAE (Fungus)				
Ustilago maydis (D. C.) Corda, MG 361	Huitlacoche	Food	All tissues	Cooked
VERBENACEAE				
Glandularia bipinnatifida (Schauer) Nutt., MG 464	Moradilla	Alopecia	Complete plant	Boiled, mixed with *Morus celtidifolia* and *Fraxinus 46uspidate* leaves, wash the head with the solution
Lantana camara L., MG 441	Lantana	Ornamental	Complete plant	Planted in gardens
Lippia graveolens Kunth, MG 442	Pionia	Asthma (bronchitis)	Leaves	Boiled, drink as tea
		Kidney	Leaves	Boiled, drink as

		diseases		tea
		Matrix inflamated	Leaves	Boiled, drink as tea
		Headache	Root	Ground, boiled, drink the solution
		Rheumatism	Root	Ground, boiled, drink the solution
Poliomintha longiflora A. Gray, MG 362	Orégano	Expectorant	Leaves	Boiled, drink as tea
		Respeiratory diseases	Leaves	Boiled, drink as tea
		Laryngitis	Leaves	Boiled, drink as tea
		Flu	Leaves	Boiled, drink as tea
		Cough	Leaves	Boiled, drink as tea
		Condiment	Leaves	Boiled, drink as tea
Verbena carolina L., MG 443	Verbena	Migraine	Branches	Boiled, drink as tea
		Skin diseases	Branches	Boiled, drink as tea
		Grains	Branches	Boiled, drink as tea
		High fever	Branches	Boiled, drink as tea
		Typhoid	Branches	Boiled, drink as tea
		Varicella	Branches	Boiled, as poultice, over the body
		Constipation	Branches	Boiled, as poultice in the stomach or, in children, mixed with *Agave lophantha* root and *Borago officinalis* leaves, blend and strain the mixture, introduce the remnants anally

				(colonic)
		Ceremonial	Complete plant	Bouquets
Verbena officinalis L., MG 444	Santa Isabel	Forage	Complete plant	Dried, raw
VIOLACEAE				
Viola tricolor L., MG 463	Violeta, pensamientos	Ornamental	Complete plant	Planted in gardens
VISCACEAE				
Phoradendron villosum (Nutt.) Nutt. ex Engelm., MG 445	Injerto	Veterinary	Leaves and branches	Boiled, mixed with salt, drink the solution to the goats to shed the remnants of the placenta
VITACEAE				
Vitis cinerea (Engelm.) Engelm. ex Millardet, MG 462	Uva cimarrona, uva de monte	Food	Fuits	Grapes, raw
		Ornamental	Complete plant	Planted in gardens
ZYNGIBERACEAE				
Zingiber officinale Roscoe, MG 528	Jenjibre or jalapeñas	Gastritis	Branches	Boiled, drink as tea
ZYGOPHYLLACEAE				
Larrea tridentata (Sessé & Moc. ex DC.) Coville, MG 286	Gobernadora	Stomach ache	Leaves	Boiled, Put 5-6 leaves in a quart of boiling water for 20 seconds (is very strong), drink as tea
		Kidney diseases	Leaves	Boiled, Put 5-6 leaves in a quart of boiling water for 20 seconds (is very strong), drink as tea
		Constipation	Leaves	Boiled, Put 5-6 leaves in a quart of boiling water for 20 seconds (is very strong), drink as tea
		Smelly feet		

		Antifungal	Leaves	Ground, use as talc, inside shoes or spreading feet
		Car cleaning radiators	Leaves and branches	Put branches and leaves in water for a day, pour it into the radiator
		Drain cleaning	Leaves and branches	Put branches and leaves in water for a day, pour it into the radiator
